# G Protein-Coupled Receptor 15 Expression Is Associated with Myocardial Infarction

**DOI:** 10.3390/ijms24010180

**Published:** 2022-12-22

**Authors:** Tina Haase, Christian Müller, Bastian Stoffers, Philipp Kirn, Melanie Waldenberger, Frank J. Kaiser, Mahir Karakas, Sangwon V. Kim, Svenja Voss, Philipp S. Wild, Karl J. Lackner, Jonas Andersson, Stefan Söderberg, Diana Lindner, Tanja Zeller

**Affiliations:** 1 University Heart & Vascular Center Hamburg, Department of Cardiology, 20246 Hamburg, Germany; 2 German Centre for Cardiovascular Research (DZHK), Partner Site Hamburg, Kiel, Lübeck, 20246 Hamburg, Germany; 3 Department of Experimental Pharmacology and Toxicology, University Medical Center Hamburg-Eppendorf, 20246 Hamburg, Germany; 4 Research Unit Molecular Epidemiology, Helmholtz Zentrum München, 85764 Neuherberg, Germany; 5 Institute of Epidemiology, Helmholtz Zentrum München, German Research Center for Environmental Health, 85764 Neuherberg, Germany; 6 German Centre for Cardiovascular Research (DZHK), Partner Site Munich, 85764 Neuherberg, Germany; 7 Institute of Human Genetics, University Hospital Essen, University Duisburg-Essen, 45147 Essen, Germany; 8 Department of Intensive Care Medicine, University Medical Center Hamburg-Eppendorf, 20246 Hamburg, Germany; 9 Department of Microbiology and Immunology, Sidney Kimmel Medical College, Thomas Jefferson University, Philadelphia, PA 19107, USA; 10 Preventive Cardiology and Preventive Medicine, Department of Cardiology, University Medical Center of the Johannes Gutenberg University Mainz, 55101 Mainz, Germany; 11 Center for Thrombosis and Hemostasis, University Medical Center of the Johannes Gutenberg University Mainz, 55101 Mainz, Germany; 12 German Centre for Cardiovascular Research (DZHK), Partner Site Rhine-Main, 55101 Mainz, Germany; 13 Institute of Clinical Chemistry and Laboratory Medicine, University Medical Center of the Johannes Gutenberg University Mainz, 55101 Mainz, Germany; 14 Department of Public Health and Clinical Medicine, Skellefteå Research Unit, Umeå University, 931 86 Skellefteå, Sweden; 15 Department of Public Health and Clinical Medicine, Umeå University, 901 87 Umeå, Sweden; 16 University Center of Cardiovascular Science, University Heart & Vascular Center Hamburg, 20246 Hamburg, Germany

**Keywords:** cardiovascular disease, G protein-coupled receptor, inflammation, biomarkers, signal pathway, gene expression, pathogenesis, epigenetics, novel molecular target, translational research

## Abstract

Beyond the influence of lifestyle-related risk factors for myocardial infarction (MI), the mechanisms of genetic predispositions for MI remain unclear. We sought to identify and characterize differentially expressed genes in early-onset MI in a translational approach. In an observational case–control study, transcriptomes from 112 early-onset MI individuals showed upregulated G protein-coupled receptor 15 (*GPR15*) expression in peripheral blood mononuclear cells compared to controls (fold change = 1.4, *p* = 1.87 × 10^−7^). *GPR15* expression correlated with intima-media thickness (β = 0.8498, *p* = 0.111), C-reactive protein (β = 0.2238, *p* = 0.0052), ejection fraction (β = −0.9991, *p* = 0.0281) and smoking (β = 0.7259, *p* = 2.79 × 10^−10^). The relation between smoking and MI was diminished after the inclusion of *GPR15* expression as mediator in mediation analysis (from 1.27 (*p* = 1.9 × 10^−5^) to 0.46 (*p* = 0.21)). The DNA methylation of two *GPR15* sites was 1%/5% lower in early-onset MI individuals versus controls (*p* = 2.37 × 10^−6^/*p* = 0.0123), with site CpG3.98251219 significantly predicting risk for incident MI (hazard ratio = 0.992, *p* = 0.0177). The nucleotide polymorphism rs2230344 (C/T) within *GPR15* was associated with early-onset MI (odds ratio = 3.61, *p* = 0.044). Experimental validation showed 6.3-fold increased *Gpr15* expression in an ischemic mouse model (*p* < 0.05) and 4-fold increased *Gpr15* expression in cardiomyocytes under ischemic stress (*p* < 0.001). After the induction of MI, *Gpr15^gfp/gfp^* mice showed lower survival (*p* = 0.042) and deregulated gene expression for response to hypoxia and signaling pathways. Using a translational approach, our data provide evidence that GPR15 is linked to cardiovascular diseases, mediating the adverse effects of smoking.

## 1. Introduction

Cardiovascular diseases (CVD) are a significant global burden and a main cause of death worldwide [1]. The leading condition of the global CVD burden is MI, which is most commonly induced by atherosclerosis [2]. As a chronic inflammatory disease of the arteries, atherosclerosis progresses slowly throughout an individual’s lifetime, and lifestyle factors, such as smoking, diet and physical inactivity, promote its progression [3,4,5]. Therefore, MI in young adults is a rare phenomenon compared to patients older than 50 years [6]. Traditional cardiovascular risk factors, such as diabetes, hypertension and hypercholesterolemia as well as a family history of ischemic heart disease and obesity, are more prevalent in early-onset MI subjects [7,8] and a specific genetic background is likely in those patients [9].

In recent years, the application of genome-wide association studies (GWAS) has led to the discovery of more than hundred genetic loci contributing to the risk of CVD [10]. However, a substantial proportion of the CVD heritability is still unexplained and the functional role of most GWAS loci is unknown [10]. To date, only a few variants have been found that specifically explain the risk of premature CVD [11] and explanations about the molecular mechanisms of the genetic predisposition remain elusive [12].

G protein-coupled receptors (GPCRs) are important cell surface receptors, mediating signals between cells and environment. More than 200 GPCRs are expressed in the heart, where they are of prime importance for cardiovascular homeostasis, regulating essential functions, such as contractility, heart rate and vascular tone [13,14,15]. Despite many GPCRs being involved in CVD, only about 4% of all GPCRs are targeted by clinical therapeutics [13]. Amongst them are important cardiovascular drugs, such as β-blockers, angiotensin II receptor blockers and angiotensin-converting enzyme inhibitors [15]. Several GPCRs have already been linked to cardiovascular risk factors, such as the *GPR15* gene (encoding the G protein-coupled receptor 15), whose DNA methylation sites showed strong association with smoking [16,17], indicating a regulation of *GPR15* gene expression by smoking. The further identification and characterization of GPCRs will likely promote the discovery of novel regulatory mechanisms and bears the potential for new (cardiovascular) drug targets and biomarkers. To date, no GPCR is known as a potential drug target or biomarker for early-onset MI. To our knowledge, no large-scale transcriptome profiling has been performed in early-onset MI individuals so far.

**Figure 1:**
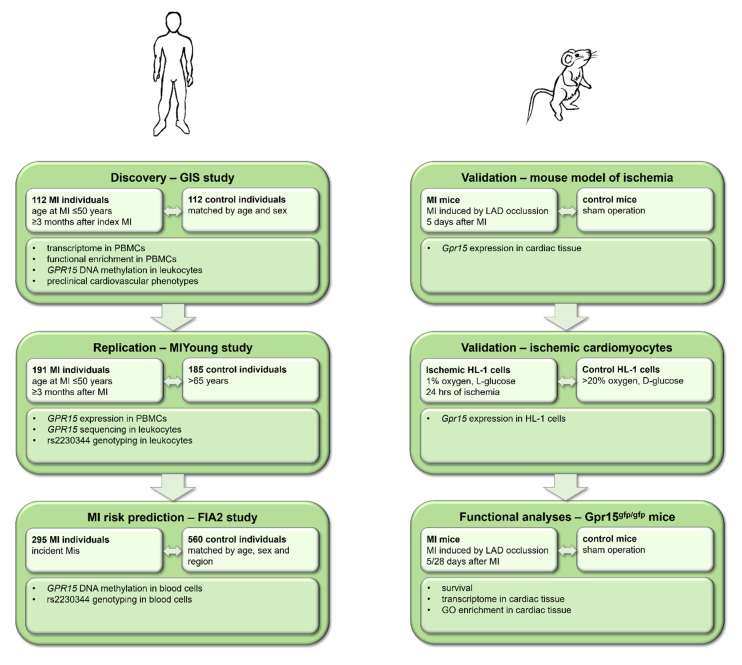
Study workflow including data from human studies and mouse experiments. In a translational approach, human data from the GIS and MIYoung studies were used for the discovery and replication of results, whereas data from the FIA2 study were analyzed for myocardial infarction (MI) risk prediction. Experimental validation and functional analyses were performed in mouse models of ischemia, ischemic cardiomyocytes and *Gpr15^gfp/gfp^* mice. PBMCs = peripheral blood mononuclear cells.

The prespecified aims of this study were to (i) identify target transcripts for MI (discovery), (ii) to independently validate the target (replication), (iii) to experimentally functionally characterize the role of the target in MI and (iv) to evaluate the potential for MI risk prediction of the target. Hence, the present study combined transcriptomic analyses in early-onset MI individuals (aged < 50 years at index event) and healthy controls with experimental analyses of mouse models, cell culture models and functional molecular analyses on the level of DNA methylation in a translational approach (Figure 1).

## 2. Results

### 2.1. Expression of GRP15 Is Increased in Early-Onset Myocardial Infarction

To identify transcripts associated with early-onset myocardial infarction on the level of mRNA, a microarray transcriptomics analysis was conducted in a discovery cohort (GIS) and validated by TaqMan qPCR in an independent cohort (MIYoung). In both the discovery and validation cohort, mRNA isolated from PBMCs was used to perform gene expression analyses.

In the discovery cohort, gene expression data of 112 early-onset MI individuals and 112 age and sex-matched healthy controls were compared using a case–control approach. The characteristics of the GIS cohort are provided in Table S1. Median age at the index event was 43 years for subjects suffering early-onset MI. Controls had a lower prevalence of classical cardiovascular risk factors, such as diabetes, obesity, hypertension and a family history of MI and higher LDL-cholesterol levels.

**Figure 2:**
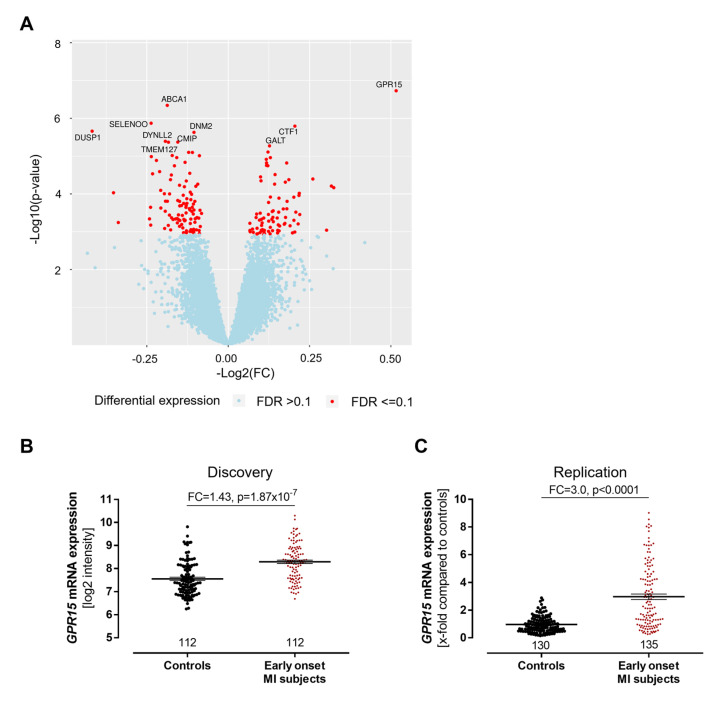
Increased *GPR15* expression after myocardial infarction. (**A**) In a transcriptome analysis of peripheral blood mononuclear cells (PBMCs) from 112 early-onset myocardial infarction (MI) patients and 112 age- and gender-matched controls from the GIS discovery cohort, *GPR15*, *ABCA1*, *SELO*, *CTF1* and *DUSP1* were the top differentially expressed genes with *GPR15* that displayed the highest fold change and lowest *p*-value. Linear mixed model adjusted for hypertension, smoking status, body mass index, diabetes and low-density lipoprotein/high-density lipoprotein ratio. FDR = false discovery rate using the q-value method. (**B**) In the GIS discovery cohort, *GPR15* mRNA expression in PBMCs was increased 1.4-fold in early-onset MI individuals (<50 years) compared to sex- and age-matched controls as determined by microarray. Linear mixed regression model adjusted for hypertension, smoking status, body mass index, diabetes and low-density lipoprotein/high-density lipoprotein ratio and technical covariates. (**C**) Increased *GPR15* expression was validated by TaqMan qPCR in the MIYoung replication cohort in PBMCs from early-onset MI individuals compared to healthy controls over 65 years. Mann–Whitney U test. Data are shown as mean ± standard error of the mean.

Out of 27,068 transcripts, 183 transcripts were differentially expressed between early-onset MI individuals and controls, with 82 transcripts being upregulated and 101 transcripts being downregulated in the early-onset MI group (Figure 2A, Table S2). Among all genes analyzed, *GPR15* (encoding the G protein-coupled receptor 15) showed the highest differential expression with a fold change of 1.43 (*p* = 1.87 × 10^−7^, Figure 2B).

Functional enrichment analysis was performed to investigate the involvement of differentially expressed genes in biological pathways. Consistently, pathway analysis revealed key cardiovascular and inflammatory signaling processes, driven by G protein signaling, to be affected in individuals with early-onset MI. The top pathways were (i) the P2Y purigenic receptor signaling pathway (8 out of 125 pathway genes), (ii) the GNRH signaling pathway (8 out of 135 pathway genes) and (iii) the renin–angiotensin signaling pathway (7 out of 113 pathway genes), indicating the important role of G protein signaling in the pathophysiology of MI.

The validation of these findings was performed by TaqMan qPCR in an independent cohort study of early-onset MI subjects, the MIYoung study, including 191 early-onset MI subjects and 185 healthy controls. The study characteristics of the MIYoung cohort are provided in Table S3. Similar to the results from our discovery cohort, *GPR15* expression was higher in early-onset MI individuals compared to the controls (3.0-fold, *p* < 0.0001, Figure 2C).

### 2.2. Relation of GPR15 Expression with DNA Methylation, Myocardial Infarction and Smoking

**Figure 3:**
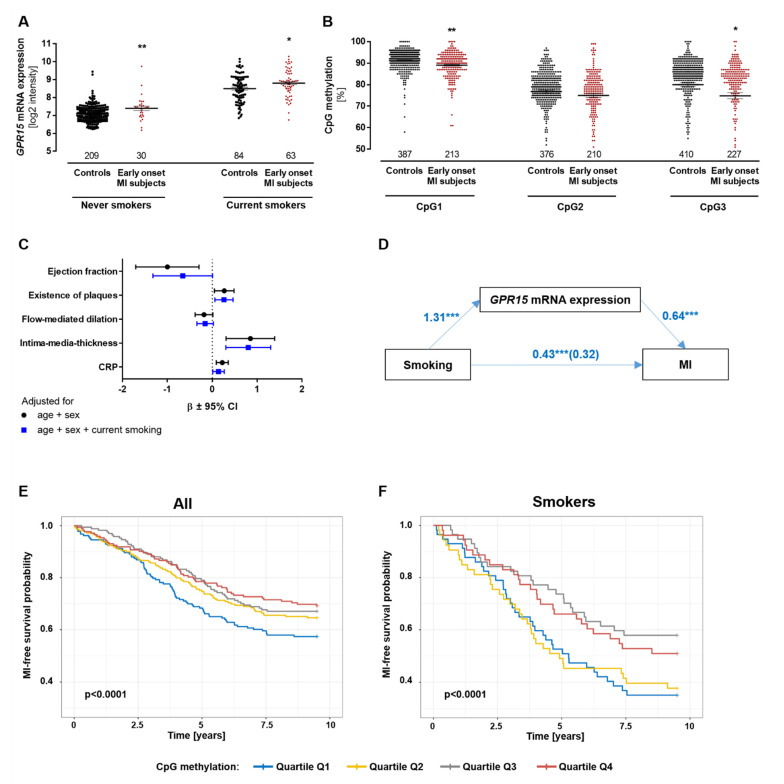
Risk for myocardial infarction predicted by *GPR15* DNA methylation independent of smoking status. (**A**) In the GIS discovery cohort, early-onset MI subjects showed significantly higher *GPR15* mRNA expression levels in BPMCs compared to controls, independent of smoking status. Mann–Whitney U test. (**B**) DNA methylation in leukocytes was 2–5% lower in MI cases compared to controls (Cpg1 *p* = 0.0021/*p* = 2.37 × 10^−6^, CpG3 *p* = 0.0201/*p* = 0.123, both adjusted for smoking). Linear mixed models adjusted for age/sex; Bonferroni correction, * = *p* < 0.05, ** = *p* < 0.01 compared to respective controls; mean ± standard error of the mean. (**C**) Correlation between *GPR15* expression in PBMCs and preclinical cardiovascular phenotypes. Intima-media-thickness was significantly associated with *GPR15* expression after adjusting for current smoking status (*p* = 0.0086). *n* = 234, linear mixed model, significance after Bonferroni correction, CRP = C-reactive protein (log scale), β = standardized beta coefficient, CI = confidence interval. (**D**) The effect of current smoking on MI was fully mediated by *GPR15* expression, as the coefficient changed from 1.27 (significant *p* = 1.9 × 10^−5^) to 0.46 (non-significant *p* = 0.21) when including *GPR15* expression. The total effect of current smoking mediated by *GPR15* expression was 0.43 (*p* < 2 × 10^−16^; average direct effects = 0.32, *p* < 2 × 10^−16^) and the unstandardized indirect effect was 0.11 (95% CI: 0.057–0.18, *p* < 2 × 10^−16^); 1000 simulations with bootstrapping).*** = *p* < 0.001 (**E**,**F**) In the FIA2 study with incident MI cases and matched controls, MI-free survival probability was significantly higher in subjects with high versus low methylation of the *GPR15* DNA methylation site CpG3 in all samples (**E**) and current smokers only (**F**) as visualized in quartiles and log-rank tests.

As previously described, *GPR15* mRNA expression and *GPR15* DNA methylation significantly associate with smoking and with each other [17,18]. The *GPR15* DNA methylation sites CpG1, CpG2 and CpG3 are located within the coding sequence of the *GPR15* gene (Figure S1). To analyze whether the changes of *GPR15* expression in early-onset MI subjects are affected by smoking, the expression of *GPR15* in PBMCs was compared between early-onset MI subjects and controls—stratified by smoking status in the GIS study. *GPR15* showed higher expression in the MI individuals (*p* = 0.004 for never smokers and *p* = 0.024 for current smokers, Figure 3A). In MI individuals, not only was *GPR15* mRNA increased but leukocytic *GPR15* DNA methylation was also significantly lower compared to controls (*p* = 0.0021 for CpG1 and *p* = 0.0504 for CpG3, Figure 3B). Even after adjusting for smoking, the DNA methylation of CpG1 and CpG3 was significantly different between MI individuals and controls (*p* = 2.4 × 10^−6^ and *p* = 0.0123, respectively). Furthermore, in early-onset MI individuals, *GPR15* was not only associated to MI but also to preclinical cardiovascular phenotypes, including ejection fraction, intima-media thickness and CRP as a surrogate marker for inflammation. The correlation to intima-media-thickness remained significant after adjustment for smoking (β = 0.8021, *p* = 0.0086, Figure 3C). To assess whether the observed association between smoking and MI was mediated by *GPR15*, causal mediation analysis was performed in the GIS-GHS study (Figure 3D). The effect of smoking on MI was fully mediated for current smokers and partially mediated for ex-smokers by *GPR15* mRNA expression (coefficients changed from 1.27 (*p* = 1.9 × 10^−5^) to 0.46 (*p* = 0.21) and from 1.64 (*p* = 1 × 10^−8^) to 1.27 (*p* = 4.3 × 10^−5^), respectively, when including *GPR15* mRNA expression to the linear model). The regression of smoking to *GPR15* mRNA expression (coefficient for current smoking 1.31, *p* < 2 × 10^−16^; coefficient for ex-smokers 0.74, *p* = 2.7 × 10^−10^) and *GPR15* mRNA expression to MI (coefficient 0.64, *p* = 2.8 × 10^−4^) were significant. The total effect of smoking mediated by *GPR15* mRNA expression was 0.43 (*p* < 2 × 10^−16^) and the unstandardized indirect effect was 0.11 (95% CI: 0.057–0.18, *p* < 2 × 10^−16^), i.e., 26.2% of the total (95% CI 13–51%).

### 2.3. GPR15 DNA Methylation Can Predict Risk of Myocardial Infarction

Based on the finding that the *GPR15* DNA methylation of sites CpG1 and CpG3 associates with early-onset MI, MI risk prediction properties were calculated for *GPR15* DNA methylation levels and incident MI individuals. Associations between the percentages of *GPR15* DNA methylation and incident MI were analyzed in the prospective nested case–control FIA2 study (comprising MI individuals and matched controls; FIA2 study characteristics are given in Table S4). The *GPR15* DNA methylation of CpG1 and CpG3 was significantly lower in current smokers compared to never smokers and ex-smokers (Figure S2), validating previous results [17]. CpG3 methylation significantly predicted the risk for incident MI after adjustment for smoking (HR = 0.992, *p* = 0.0177, Table S5, Figure S3). As shown in Kaplan–Meier curves in Figure 3E/F, MI risk was lower in individuals with higher CpG3 DNA methylation levels compared to individuals with lower CpG3 DNA methylation levels. This was shown in the overall cohort (Figure 3E) as well as in smokers only (Figure 3F; *p* < 0.0001). Hence, the probability of MI-free survival was higher in individuals with higher CpG3 DNA methylation levels compared to individuals with lower CpG3 DNA methylation levels.

### 2.4. GPR15 SNP rs2230344 Associates with Early-Onset Myocardial Infarction

To identify genetic variants linked to *GPR15* expression and myocardial infarction, the entire *GPR15* gene was sequenced in a subgroup of early-onset MI individuals and healthy controls from the MIYoung study. The subgroup consisted of *n* = 32 individuals having had an MI at an age below 40 years. The characteristics of this subgroup of early-onset MI individuals and controls (*n* = 32) are given in Table S6. The single nucleotide polymorphism (SNP) rs2230344 (C/T) showed a minor allele frequency (T) in early-onset MI individuals of 9% compared to 22% in healthy controls (OR = 3.74, *p* = 0.048 for number of C alleles; adjusted for BMI). Validation by qPCR showed consistent results, with a rs2230344-T minor allele frequency of 13% for early-onset MI individuals and 23% for healthy controls (OR = 3.61, *p* = 0.044 for number of C alleles; adjusted for BMI).

rs2230344 is located in close proximity to the *GPR15* DNA methylation sites (Figure S1, USCS Genome Browser [19]) and thus might influence *GPR15* mRNA expression or DNA methylation. However, no significant association was shown between rs2230344 and *GPR15* mRNA expression or DNA methylation in the MIYoung study (Figure S4, Table S7) nor did the rs2230344 minor T allele predict the MI risk in the FIA2 study (Table S8).

### 2.5. Experimental Validation of Increased Gpr15 Expression after Ischemia

**Figure 4:**
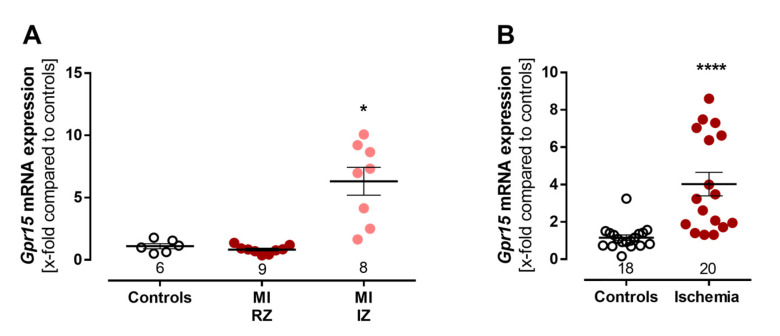
Experimental validation of increased *Gpr15* expression after induced myocardial infarction. (**A**) Validation of increased *Gpr15* expression in a mouse model of myocardial infarction. Cardiac *Gpr15* expression was increased 6.3-fold in the infarct zone (IZ), but not in the remote zone (RZ), five days after MI in male C57BL/6J (B6) mice. Kruskal–Wallis test with Dunn’s multiple comparisons test. (**B**) Validation of increased cardiac *Gpr15* expression in a cell culture model of ischemic cardiomyocytes. HL-1 cells were incubated with 20% oxygen/D-glucose (control) and 1% oxygen/L-glucose (ischemia) for 24 h. *Gpr15* expression was increased 4-fold in ischemic cardiomyocytes (*n* = 4 experiments). Mann–Whitney U test. Expression levels were normalized to the reference 18S rRNA and to the expression levels of the control group and plotted as x-fold expression using the formula 2^−ΔΔCt^. * = *p* < 0.05, **** = *p* < 0.0001 compared to the respective control group. Data are shown as mean ± standard error of the mean.

To assess our findings of an increased *GPR15* expression after MI in experimental models, *Gpr15* mRNA expression was measured in a mouse model of infarction-related ischemia as well as in an ischemic cell culture model. Cardiac *Gpr15* expression significantly increased five days after MI in the scared infarct zones (IZ) (6.32-fold, *p* < 0.05, Figure 4A). On cellular level, *Gpr15* mRNA expression was determined in a model of ischemic HL-1 cardiomyocytes. Here, the 24 h induction of ischemic stress significantly increased *Gpr15* mRNA expression 4-fold in a cell culture model of ischemic cardiomyocytes compared to controls (*p* < 0.001, Figure 4B), affirming the increased cardiac *Gpr15* expression in infarcted mouse hearts.

### 2.6. Gpr15 Knockout Affects Survival and Cardiac Remodeling in a Murine Model of Myocardial Infarction

**Figure 5:**
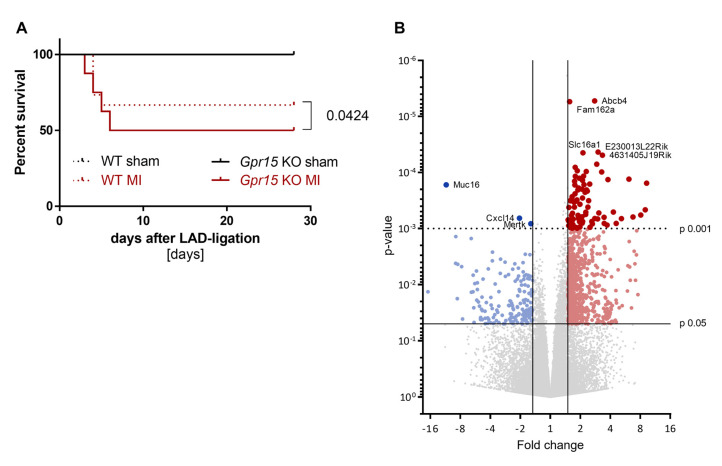
Reduced survival and differential gene expression in *Gpr15^gfp/gfp^* mice after induced myocardial infarction. (**A**) Survival was slightly lower in *Gpr15^gfp/gfp^* mice compared to wildtype mice (*p* = 0.0424, *n* = 5/6 sham and 15/16 MI). log-rank test (**B**) Gene expression measured by 3′ mRNA sequencing of cardiac tissue from the IZ from nine Bl6 wildtype (WT) and eight *Gpr15^gfp/gfp^* mice 5 days after MI surgery. Downregulated genes in cardiac tissue from *Gpr15^gfp/gfp^* compared to WT mice are depicted in blue, while red highlights upregulated genes. Fold change and *p*-value are displayed in the volcano plot to identify significant differences in gene expression. Further restricted by *p*-value ≤ 0.001, 84 top differentially expressed genes were identified (81 upregulated and three downregulated genes). Gene symbols for five upregulated genes with the lowest *p*-value and for the downregulated genes are displayed. Red and blue colors highlight significantly regulated genes with *p*-value < 0.05 (small dots) or *p*-value < 0.001 (large dots).

In humans, we showed increased *GPR15* expression after myocardial infarction, combined with altered G protein signaling pathways. To characterize the role of Gpr15 in MI and to identify molecular pathways through which Gpr15 acts in the heart, *Gpr15^gfp/gfp^* mice were subjected to MI induced by LAD ligation (Figure S5). *Gpr15^gfp/gfp^* mice did not show significant hemodynamic differences compared to wildtype (WT) mice. However, Kaplan–Meier survival curves revealed a significantly lower survival 28 days post MI in *Gpr15^gfp/gfp^* compared to WT mice (*p* = 0.0424, Figure 5A).

**Figure 6:**
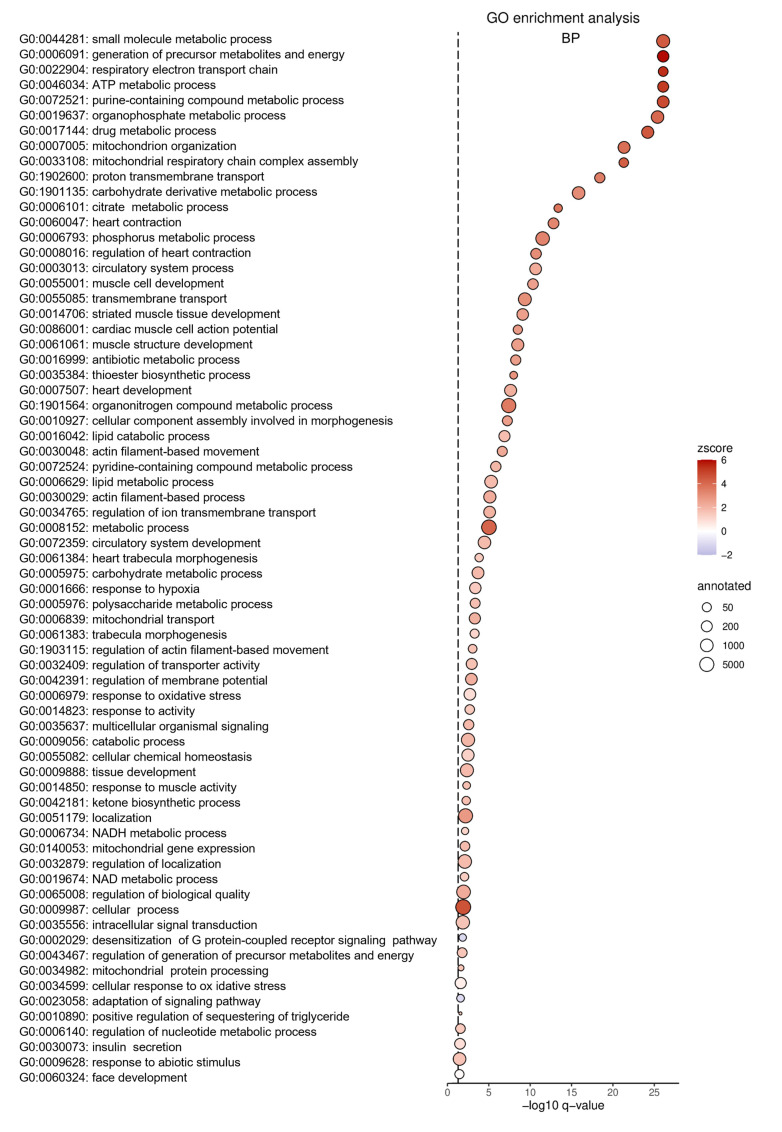
Altered GO terms in *Gpr15^gfp/gfp^* mice after induced myocardial infarction. Significant gene ontology (GO) terms for biological processes (BP) clustered utilizing Revigo. Differentially expressed GO terms for *Gpr15^gfp/gfp^* mice include *response to hypoxia*, *desensitization of GPCRs* and *intracellular signal transduction*. The GO term ID and the description were plotted according to their *p*-value as dot plot. On the *x*-axis, the negative decadic logarithm of the *p*-value of the GO terms is shown. The individual GO terms are displayed on the *y*-axis, sorted according to their *p*-value. The size of the dots represents the number of all genes that are annotated in the respective GO term. The color of the dots is based on the GO term’s z-score. Whether the majority of genes in a GO term is up- or downregulated is indicated by the color gradient from red (upregulated) to blue (downregulated), respectively.

To identify molecular pathways affected by Gpr15, RNA from cardiac tissue from the IZ from WT and *Gpr15^gfp/gfp^* mice 5 days after MI was analyzed by 3’ mRNA sequencing. In the IZ, 822 genes were identified as significant differentially expressed (*p* < 0.05) between *Gpr15^gfp/gfp^* and WT mice (Figure 5B, Table S9). Out of these 822 genes, 656 were significantly upregulated (red) in *Gpr15^gfp/gfp^* mice and 166 genes were significantly downregulated (blue). The top differentially expressed genes were *Myl7*, *Myl4* (upregulation) and *Epo* and *Ccrl2* (downregulation). The resulted GO term analyses for biological processes that were differentially regulated in the IZ are plotted in Figure 6, the GO term analyses for molecular functions and for cellular components are plotted in Figures S6 and S7, respectively. The identified GO terms affected by Gpr15 knockout include *desensitization of GPCR*, *response to hypoxia*, *intracellular signal transduction*, *adaptation of signaling pathway* and *multicellular organismal signaling* (Figure 6).

## 3. Discussion

Understanding the molecular mechanisms of CVD is of great importance for the identification of novel biomarkers and the development of therapeutic targets for CVD. In this study, transcriptomic analyses in early-onset MI individuals were performed to identify novel genes related to CVD. The G protein-coupled receptor 15 had previously been implicated to play a role in inflammatory diseases [20,21,22,23,24]. Even though the identification of GPR15 signaling, as well as the identification of GPR15 ligand-binding, has progressed significantly within the last years and GPR15 activation has been demonstrated in vascular endothelial cells, insights into the physiological and pathophysiological role of GPR15 remain scarce [25]. Here we show that (i) *GPR15* is upregulated in early-onset MI subjects; (ii) *GPR15* mRNA expression mediates the effect of smoking on MI; (iii) *GPR15* DNA methylation predicts risk for MI, independently of smoking; (iv) SNP rs2230344 is associated with early-onset MI; and v) Gpr15 knockout reduces survival after MI and affects response to hypoxia and GPCR pathways in an experimental mouse model of myocardial infarction.

To identify molecular mechanisms for CVD, we focused on RNA expression in early-onset MI individuals, being under stable disease conditions for at least three months after the index event to prevent a bias by post-acute MI inflammatory and remodeling processes. The *GPR15* gene expression was most significantly upregulated in early-onset MI individuals. Additionally, GPCR pathways were shown to be regulated in early-onset MI individuals, indicating an important role of G protein signaling in the pathophysiology of MI. Consistently, GPCRs are known to play important roles in cardiovascular function and CVD [14]. GPR15 itself is a GPCR as well, which supports its connection to CVD. Furthermore, GPR15 has been described to act as a T cell homing receptor and to be involved in inflammatory diseases [20,21,22,23,24]. The development of CVD involves chronic inflammatory processes and inflammation also plays an important role after MI [3,26]. In early-onset MI individuals, *GPR15* expression was also associated with subclinical cardiovascular phenotypes and inflammation, suggesting a potential involvement in or the potential use of GPR15 as a surrogate marker for the development of early-onset MI.

Consistent with the transcriptome analysis, the increased *GPR15* expression was replicated in a second cohort with early-onset MI individuals. The controls in the replication cohort were older than 65 years and with increasing age, the influence of genetic CVD heritability is less prominent than the influence of cardiovascular risk factors, suggesting that *GPR15* might play a role in CVD on a genetic level. Furthermore, increased cardiac *Gpr15* expression was validated in a mouse model of MI as well as in ischemic cardiomyocytes. These data indicate a specific response to conditions of MI regarding myocardial ischemia, which is in agreement with the human data. Consequently, our data imply that *GPR15* might play a role in the pathogenesis of acute MI and in the conditions of ischemia, such as artery narrowing by plaques.

Previously, we described a substantial association between smoking, *GPR15* DNA methylation and *GPR15* mRNA expression [17]. DNA methylation within the gene body, as measured in the current study, has been described in relation to mRNA expression levels [27,28]. Specifically, for *GPR15*, tobacco smoking seems to alter blood cell composition, thereby affecting *GPR15* expression levels [29]. In the current study, we show not only higher *GPR15* mRNA expression but also lower *GPR15* DNA methylation in early-onset MI individuals. Smoking itself poses a strong risk factor for CVD [5]. Interestingly, the associations between *GPR15* mRNA expression and *GPR15* DNA methylation with myocardial infarction were found to be independent of smoking status. The effect of smoking on MI, however, was mediated by *GPR15* mRNA expression. Hence, our data suggest that changes in *GPR15* expression or DNA methylation and association to early-onset MI might mediate smoking effects while not excluding other unknown mechanisms.

On the genetic level, our data indicated the lower allele frequencies of the minor allele of SNP rs2230344 in early-onset MI individuals. Potentially, rs2230344 might influence the development of early-onset MI via an effect on *GPR15* DNA methylation, as previously described in African Americans [30] and/or by affecting *GPR15* expression. Our data, however, did not reveal an association between rs2230344 and *GPR15* DNA methylation. Nevertheless, rs2230344 is located within a region of the *GPR15* gene coding for a transmembrane domain. The minor T allele is a missense variant, which leads to an amino acid exchange from proline to serine. The lower abundance of the T allele in early-onset MI subjects might cause changes in protein conformation and thus the integration of the GPR15 protein into the plasma membrane or even affect GPR15 protein function.

In order to characterize the role of Gpr15 in MI and to identify underlying molecular mechanisms, we experimentally assessed the role of Gpr15 in a mouse model of MI induced by LAD. After the induction of MI, *Gpr15^gfp/gfp^* mice had reduced survival compared to wildtype mice. On a molecular level, we showed that genes deregulated in *Gpr15^gfp/gfp^* mice after MI induction belong to the GO terms desensitization of GPCR, signaling pathways (such as the *adaptation of signaling pathway*, *intracellular signal transduction*) and response to hypoxia pathways. Similar to the molecular mechanisms in the *Gpr15^gfp/gfp^* mice, in the human early-onset MI transcriptome analysis, GPCR signaling pathways were affected. Taken together, these mouse data indicate that Gpr15 might be involved in signaling processes in response to hypoxia after MI, potentially explaining why *Gpr15^gfp/gfp^* mice showed reduced survival. Given the level of significance, the relevance and reproducibility of the reduced survival rate has to be elucidated in further studies.

In our study, Gpr15 was involved in immune response mechanisms in a mouse model of MI. Consistently, our human data showed an association between *GPR15* mRNA expression with CRP as a marker of inflammation. This is in line with previous human and mouse studies, which show an involvement of GPR15 in inflammatory diseases, such as inflammatory bowel disease and rheumatoid arthritis [20,21]. Whether or not, and under which conditions, GPR15 could have pro- or anti-inflammatory properties, possibly varying between conditions and species [31], is still to be determined and requires further investigations.

Our presented work comprises large-scale human data, including longitudinal data and different omics data, combined with experimental models. Unique data from early-onset prevalent and incident MI individuals showed changes in *GPR15* mRNA expression as well as *GPR15* DNA methylation and rs2230344 allele frequency. The DNA methylation of the *GPR15* CpG3 site even predicted the MI risk. Nevertheless, limitations include sample availability, ethnicity and effect sizes. Human samples were available from Caucasian individuals. Ethnicity largely affects molecular mechanisms, e.g., as the association between the SNP rs2230344 genotype and *GPR15* DNA methylation has previously been reported for African Americans but not for Caucasians [30]. As only DNA but no RNA is available in the FIA2 study, the prediction of MI risk for *GPR15* mRNA levels was not possible. Changes in mRNA expression and DNA methylation might further be caused by variations in cell composition; however, no data on cell composition has been measured and is thus not available. Even though gene expression and DNA methylation changes are of small effect sizes, they are significant and persistent throughout human and mouse data. In mice, Gpr15 expression was increased in ischemic cardiac tissue. A possibility of evaluating the involvement of Gpr15 in the pathogenesis of MI, a combination of an atherosclerosis mouse model (e.g., apolipoprotein E knockout mice) and Gpr15 knockout mouse model might be suitable.

## 4. Materials and Methods

The present study combined transcriptomic analyses in early-onset MI individuals (aged < 50 years at index event) and healthy controls with experimental analyses of mouse models, cell culture models and functional molecular analyses on the level of DNA methylation in a translational approach. The study workflow is shown in Figure 1.

### 4.1. Human Cohorts

All human cohorts followed the Declaration of Helsinki. All subjects gave written informed consent. Detailed cohort descriptions are shown in Table S10.

#### 4.1.1. Gutenberg Young Myocardial Infarction Study (GIS)

Caucasian individuals with a history of early-onset MI (≤50 years) were recruited into the Gutenberg Young Myocardial Infarction Study (GIS). Individuals were invited to participate in GIS according to the following criteria: (i) confirmed MI (positive ECG, increase in biomarkers of cardiac necrosis (troponin, creatine-kinase MB) or clinical symptoms) and proven by coronary angiography, (ii) age at myocardial infarction ≤50 years (as described previously [32]) and (iii) stable disease conditions for at least three months before study participation. The study protocols and sampling design were approved by the local ethics committee of the Medical Chamber of Rhineland-Palatinate, Germany (ethical approval code 837.211.08 (6208)). Caucasian individuals without manifest cardiovascular disease and recruited as part of the Gutenberg Health Study (GHS) [33,34] were used as controls. Out of 234 MI patients and 419 controls, 112 case-control pairs were matched by age and gender. The study protocols and sampling design were approved by the local ethics committee of the Medical Chamber of Rhineland-Palatinate, Germany (ethical approval code 837.020.07 (5555)).

The participants of GIS and GHS visited the same study center simultaneously. Self-reported smoking status was classified as follows: current smokers (including occasional smokers), ex-smokers (smoking cessation at least six weeks before study participation) and never smokers. Cumulative smoking exposure was evaluated by pack years (one pack year = smoking of 20 cigarettes per day for one year) for current smokers and ex-smokers. Peripheral blood mononuclear cells (PBMC) RNA was isolated via Trizol extraction and leukocytic DNA was isolated as described by Miller et al. from buffy-coated ethylenediamine tetra-acetic acid blood samples [35,36].

#### 4.1.2. Young Myocardial Infarction Study (MIYoung)

The MIYoung study is a clinical cohort study from the University Heart and Vascular Center Hamburg, Medical University Hamburg-Eppendorf, including subjects with a history of early-onset MI at an age < 50 years and under stable disease conditions for at least three months (*n* = 191) and healthy controls over 65 years (*n* = 185). The study protocols and sampling design were approved by the local ethics committee of the University Medical Center Hamburg-Eppendorf, Germany (ethical approval code PV4137). Self-reported smoking status was classified as follows: current smokers, ex-smokers (smoking cessation at least six weeks before study participation) and never smokers. From all participants, PBMC RNA and leukocytic DNA was isolated.

#### 4.1.3. First-Ever Myocardial Infarction Study 2 (FIA2)

The FIA 2 study is a prospective nested case-referent study from Northern Sweden, including incident MI cases matched with two healthy controls for age, sex, date of health examination and geographical region [37]. Subjects were recruited from the Northern Sweden MONitoring of Trends and Determinants in CArdiovascular Diseases (MONICA) project, the Västerbotten Intervention Program (VIP), and the Mammary Screening Program (MSP). MI events were identified through the screening of hospital discharge records, general practitioners’ reports and death certificates [38]. Smoking status was classified as smokers (daily smokers), ex-smokers and non-smokers. Blood samples obtained at the baseline health survey were used, and sampling was carried out 3.9 years (interquartile range 3.6 years) before the MI event. DNA was isolated by phenol-chloroform extraction or salting-out precipitation. The study was approved by the local ethics committee (Dnr 05-142M, with an additional review on 19 January 2009).

### 4.2. Mouse Models

Male C57BL/6J (B6) mice at the age of 8 to 10 weeks were used for the presented animal mouse models. Maximal five mice per cage were kept in standard cages under a 12 h:12 h light:dark cycle, constant temperature and humidity and received standard food and water ad libitum. At the time of experiments, animals were six- to twelve-weeks-old. All animal investigations conform to the Guide for the Care and Use of Laboratory Animals published by the US NIH [39]. The study protocols were approved by the Hamburg Authority for Health and Consumer Protection (approval codes G060/15) and the Regional Office for Health and Social Affairs Berlin (approval code G0055/11).

*Gpr15^gfp/gfp^* mice had a GFP knock-in, resulting in a partial deletion of *Gpr15* exon 1, as described previously [20]. Male wildtype and *Gpr15^gfp/gfp^* mice were housed together and were randomly allocated by housing cages to either intervention or control group. To induce MI (intervention), mice were subjected to the permanent ligation of the left anterior descending artery (LAD). Mice of the control group underwent a sham operation, followed by recovery for five (*n* = 6/9) or 28 days (*n* = 7/16). All procedures were carried out as previously described in detail [40,41]. Briefly, mice were anesthetized with isoflurane for the surgical procedure and buprenorphine was given as analgesic therapy. The heart was accessed via the third left intercostal space and the LAD was permanently ligated with a surgical suture. Sham animals underwent the same procedure except for LAD ligation. Hemodynamic measurements were performed using a microconductance catheter system positioned in the left ventricle in closed-chest animals through the right carotid artery. After the explantation of the heart, the atria and right ventricle were removed and left ventricular tissue was dissected into post-infarction zone (IZ) and unaffected surrounding LV-tissue (remote zone, RZ), immediately snap-frozen in liquid nitrogen and stored at −80 °C. Directly after explantation, hearts were inspected for a distinctly visible infarcted area. Total RNA was isolated using QIAzol lysis reagent followed by purification using the miRNeasy mini kit (Qiagen, Hilden, Germany).

### 4.3. Cell Culture

HL-1 cells were cultivated in Claycomb medium, including 10% FBS, 1% Penicillin-Streptomycin, 2 mM GlutaMAX and 0.1 µM noradrenalin and incubated at 37 °C in a humidified atmosphere of 5% CO_2_. To simulate ischemic conditions in vitro, HL-1 cells were cultivated until confluency, starved in serum-reduced medium with 0.5% FCS overnight and placed in the hypoxic chamber. Oxygen supply was reduced from 20% to 1% and controlled with an oxygen sensor and D-glucose was replaced by L-glucose to keep osmotic properties. Cells were incubated for 24 hrs. Total RNA was isolated using the RNeasy mini kit (Qiagen, Hilden, Germany).

### 4.4. Gene Expression Analysis

Human transcriptomes from the GIS-GHS study were analyzed using the Affymetrix GeneChip Human Exon ST1.0 Array (Applied Biosystems, Waltham, MA, USA). The measurement of mouse transcriptomes by massive analysis of cDNA Ends (MACE) 3’ mRNA sequencing was conducted by GenXPro (Frankfurt, Germany). Real-time quantitative polymerase chain reaction (qPCR) with the 7900 TaqMan system (Applied Biosystems, Waltham, MA, USA) was used to determine mRNA expression levels. RNA was reverse transcribed using a High-Capacity cDNA Reverse Transcription Kit (Applied Biosystems Waltham, MA, USA), according to manufacturers’ protocols. For gene expression analyses, the *GPR15* Hs00922903_s1 (human) and *Gpr15* Mm03990531_s1 (mouse) expression assays were used (Applied Biosystems Waltham, MA, USA). The quantification of housekeepers *GAPDH* (Hs99999905_m1) or 18S rRNA (Hs99999901_s1) as internal controls was performed for each sample (Applied Biosystems Waltham, MA, USA). Data were normalized to *GAPDH* or 18S rRNA levels (∆Ct-values) to account for RNA input and relative gene expression levels were expressed in comparison to the corresponding untreated controls using the formula 2^−∆∆Ct^ [42].

### 4.5. Analyses of DNA Methylation

Previously, the DNA methylation levels of sites CpG3.98251047, CpG3.98251179 and CpG3.98251219, located within the *GPR15* gene body, were identified to differ between smokers and non-smokers [17]. The DNA methylation levels of the *GPR15* DNA methylation sites CpG3.98251047, CpG3.98251179 and CpG3.98251219 (referred to as GpC1, CpG2 and CpG3, respectively) were measured using the EpiTYPER MassARRAY technology (Agena Bioscience, Hamburg, Germany) as described previously [17,43]. The *GPR15* gene from chr3:98531454 to chr3:98533234 (GRCh38/hg38) was amplified by PCR and sequenced by Sanger sequencing. The genotyping of the rs2230344 SNP was carried out by qPCR using the Applied Biosystems C__22275108_10 TaqMan^®^ SNP Genotyping Assay on a 7900 HT Real Time PCR system (Applied Biosystems, Waltham, MA, USA).

### 4.6. Statistical and Bioinformatical Analyses

Differential gene expression between sex and age-matched groups of the GIS-GHS study was identified by applying a linear mixed model using R-package nlme [44,45]. The model was adjusted for the classical cardiovascular risk factors hypertension, smoking status, body mass index (BMI), diabetes and LDL/high-density lipoprotein (HDL) ratio. Correction for multiple testing was performed by calculating a false discovery rate (FDR) for each gene using the q-value method the Bioconductor package q value [46,47]. The significance level for differential gene expression was set to FDR < 0.1. Gene expression differences were reported as fold changes (FC), which represent the absolute expression changes corrected for covariates used in adjustment. To explore biological functions, canonical pathways and networks, functional enrichment analyses using ingenuity pathway analysis (IPA) was performed. If genes were differentially expressed with an FDR < 0.1 (*n* = 183), their respective FCs and *p*-values were uploaded into IPA. The reference set was restricted to genes on the Affymetrix Exon ST 1.0 Array.

Differential gene expression between *Gpr15^gfp/gfp^* and wildtype mice was calculated using DESeq2 [48] and plotted using Graph Pad Prism (GraphPad Software, San Diego, CA, USA). Gene ontology (GO) annotation data are based on ENSEMBL. Subsequent GO enrichment analysis was performed using the topGO package (bioconductor). The enrichment of GO terms was calculated by the Fisher’s exact test based on transcripts with a *p*-value < 0.05. Due to the large number of significant GO terms, ReviGo was used for clustering [49]. Resulting GO terms were visualized by dot plots generated with ggplot2 [50]. The z-score was calculated for each GO term by subtracting the number of downregulated genes (FC < −1.5, *p* < 0.05) from the number of upregulated genes (FC > 1.5 and *p*-value < 0.05) and dividing the result by the square root of the number of annotated genes.

Nonparametric Mann–Whitney U test and Kruskal–Wallis test with Dunn’s multiple comparisons test were used to compare two or more than two groups, respectively. Linear mixed regression models were adjusted for batch, using the random variable, and age and sex as covariates, except for the MIYoung study, where no adjustment for age was performed. Values for C-reactive protein were log-transformed. For rs2230344 allele association with early-onset MI in the MIYoung study, logistic regressions with MI as the dependent variable and rs2230344 as the independent variable of interest were performed and adjusted for BMI. To assess whether the percentage of CpG methylation levels or rs2230344 minor T allele were significantly related to increased risk of MI, mixed effect Cox models were applied using the R *coxme* package. For significant DNA methylation sites, Kaplan–Meier curves were generated and tested by log-rank tests using the R *survival* package. The survival of mice after LAD-ligation was plotted as a Kaplan–Meier curve and analyzed by log-rank test using GraphPad Prism. Causal mediation analysis in the GIS-GHS study—with smoking as the independent variable, MI as the dependent variable and *GPR15* mRNA as the potential mediator—was conducted using the R package *mediation* [51]. Unstandardized indirect effects were computed by bootstrapping samples (1000 simulations), and the 95% confidence interval (CI) as indirect effects at the 2.5th and 97.5th percentiles. Linear models for mediators and independent variables vs. MI were computed adjusting for age, sex, diabetes status, hypertension status, HDL and LDL.

Statistical analyses were performed and figures were prepared using R version 3.4.3 and GraphPad prism version 6.05 for Windows (GraphPad Software, San Diego, CA, USA) [44]. The threshold for statistical significance was set at *p* ≤ 0.05 (two-sided testing) after Bonferroni correction to adjust for multiple testing when applicable.

## 5. Conclusions

**Figure 7:**
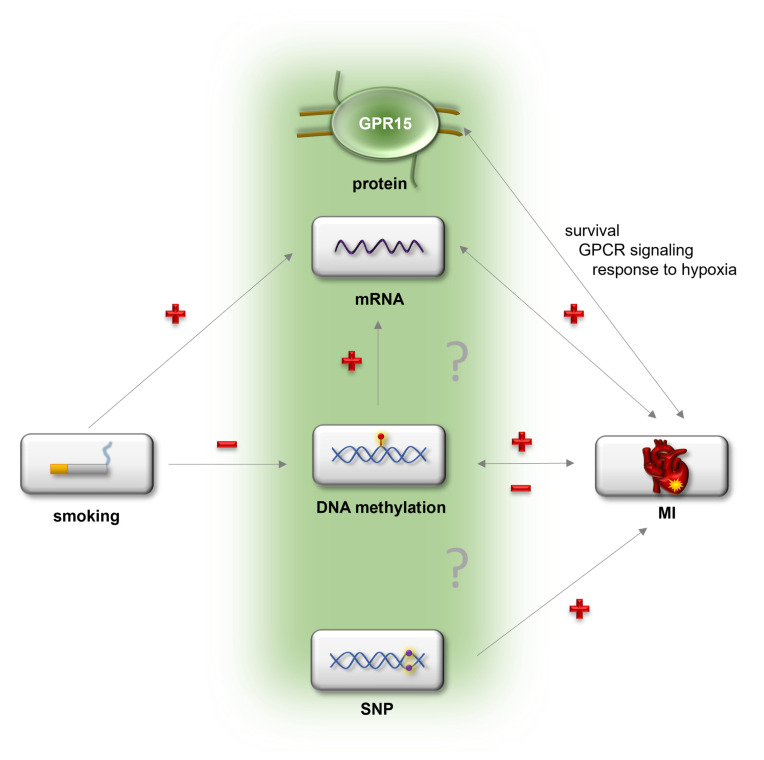
Proposed model of the potential connection between GPR15 and myocardial infarction. Possibly, smoking as a major risk factor for myocardial infarction (MI) might change blood cell composition and thereby *GPR15* mRNA expression as well as *GPR15* DNA methylation. Myocardial infarction is associated with elevated *GPR15* mRNA expression as well as decreased *GPR15* DNA methylation. The effect of smoking on MI is mediated by *GPR15* mRNA expression. Gpr15 affects survival as well as signaling and gene expression related to signaling and response to hypoxia after MI. The SNP rs2230344 within the *GPR15* gene is associated with early-onset MI. “+” = upregulation, “−” = downregulation.

In summary, for the first time this study showed an involvement of GPR15 in myocardial infarction, adding an important piece of the puzzle to understanding the physiological and pathophysiological role of GPR15. A proposed model linking of GPR15 and MI is depicted in Figure 7. Future analyses are needed to show the exact molecular pathways linking GPR15 in CVD pathogenesis and whether GPR15 might be causal for MI development. With this knowledge, GPR15 bears potential as a valuable therapeutic target for CVD prevention or treatment, in particular as protein-binding GPCRs, such as GPR15, bind only a limited number of ligands (51). As *GPR15* DNA methylation predicted MI risk and *GPR15* expression was associated with preclinical cardiovascular phenotypes, the potential of GPR15 as a potential biomarker for CVD risk prediction merits further evaluation.

## Data Availability

The genotype, transcriptomics and phenotypic data are available under restricted access, as they contain identifying participant information. Deposition in online repositories or controlled access repositories is not authorized by the patients’ consent. Access requests, which must include a formal research proposal indicating the use of data and planned analyses, should be addressed to Tanja Zeller (t.zeller@uke.de).

## Supplementary Materials

The following supporting information can be downloaded at: https://www.mdpi.com/article/10.3390/ijms24010180/s1.
