# Supplementary material for: G Protein-Coupled Receptor 15 Expression Is Associated with Myocardial Infarction
Source: Int J Mol Sci. 2022 Dec 22;24(1):180. doi: 10.3390/ijms24010180 (PMC9820726; doi:10.3390/ijms24010180)
Supplement: Supplementary file 1 [file ijms-24-00180-s001.zip › ijms-2030810-supplementary.pdf]

International Journal of *Molecular Sciences*

MDPI

Supplementary Materials

G Protein-Coupled Receptor 15 Expression Is Associated with Myocardial Infarction

Tina Haase[1,2], Christian Müller[1,2], Bastian Stoffers[1,2], Philipp Kirn[2,3], Melanie Waldenberger[4,5,6], Frank J. Kaiser[7], Mahir Karakas[2,8], Sangwon V. Kim[9], Svenja Voss[1,2], Philipp S Wild[10,11,12], Karl J. Lackner[12,13], Jonas Andersson[14], Stefan Söderberg[15], Diana Lindner[1,2#,†], Tanja Zeller[2,16*,†]

Table S1: page 2
Table S2: provided as separate Excel sheet in Supplements_2
Table S3: page 4
Table S4: page 5
Table S5: page 6
Table S6: page 7
Table S7: page 8
Table S8: page 9
Table S9: provided as separate Excel sheet in Supplements_2
Table S10: page 11

Figure S1: page 13
Figure S2: page 14
Figure S3: page 15
Figure S4: page 16
Figure S5: page 17
Figure S6: page 18
Figure S7: page 19

Table S1. Study characteristics of the early-onset MI individuals and controls of the Gutenberg Young Infarction Study.

Characteristics	All	Controls	MI cases
n (%)	224	112 (50)	112 (50)
Females, n (%)	72 (32)	36 (32)	36 (32)
Age, years	45 (42-49)	45 (41-49)	45 (42-49)
Age at MI	43 (40-48)	NA	43 (40-48)
Cardiovascular risk factors			
Diabetes mellitus, n (%)	13 (6)	2 (2)	11 (10)
Body mass index, kg/m²	27 (24-32)	26 (23-29)	29 (26-33)
Hypertension, n (%)	99 (44)	39 (35)	60 (54)
Current smoker, n (%)	61 (27)	30 (27)	31 (28)
Family history of MI, n (%)	47 (21)	10 (9)	37 (33)
Lipid status			
LDL-cholesterol, mg/dl	120 (93-146)	136 (119-162)	99 (79-121)
HDL-cholesterol, mg/dl	46 (39-55)	51 (42-58)	42 (37-52)
LDL/HDL-ratio	2.5 (1.8-3-3)	2.7 (2.2-3.4)	2.3 (1.7-3.1)

Continuous variables are described by median values (25th percentile to 75th percentile). Dichotomous variables are presented as total numbers (%). HDL = high-density lipoprotein, LDL=low-density lipoprotein, MI=myocardial infarction, NA=data not available

Table S2. Significantly differentially expressed genes between 112 early-onset MI patients and sex- and age-matched controls from the GIS discovery cohort.

Table S2 is provided at the end of this file

Table S3. Study characteristics of the early-onset MI patients and controls of the MIYoung study.

Characteristics	All	Controls	MI cases
n (%)	376	185 (49)	191 (51)
Females, n (%)	23 (6)	0 (0)	23 (12)
Age, years	65 (48-70)	70 (67-73)	48 (43-51)
Age at MI	44 (39-47)	NA	44 (39-47)
Cardiovascular risk factors			
Diabetes mellitus, n (%)	39 (10)	16 (9)	23 (12)
Body mass index, kg/m²	27 (24-29)	26 (24-28)	28 (25-31)
Hypertension, n (%)	155 (41)	66 (36)	89 (47)
Current smoker, n (%)	55 (15)	8 (4)	47 (25)
Family history of MI, n (%)	106 (28)	29 (16)	77 (40)
Lipid status			
LDL-cholesterol, mg/dl	99 (74-125)	115 (93-137)	82 (55-103)
HDL-cholesterol, mg/dl	48 (41-60)	56 (46-66)	43 (36-51)
LDL/HDL-ratio	1.9 (1.4-2.5)	2.0 (1.5-2.5)	1.8 (1.2-2.3)

Continuous variables are described by median values (25th percentile to 75th percentile). Dichotomous variables are presented as total numbers (%). HDL = high-density lipoprotein, LDL=low-density lipoprotein, MI=myocardial infarction, NA=data not available

Table S4. Study characteristics of incident MI individuals and controls of the FIA2 cohort.

Characteristics	All	Controls	MI cases
n (%)	855	560 (65)	295 (35)
Females, n (%)	146 (17)	94 (17)	52 (18)
Age, years	53 (50-60)	53 (50-60)	54 (50-60)
Age at MI	57 (52-64)	NA	57 (52-64)
Current smoker, n (%)	252 (29)	118 (21)	134 (45)

Continuous variables are described by median values (25th percentile to 75th percentile). Dichotomous variables are presented as total numbers (%). MI=myocardial infarction, NA=data not available

Table S5. Incident myocardial infarction risk predicted by *GPR15* DNA methylation sites in the FIA2 study.

DNA methylation site	Hazard ratio	95% CI	p value
CpG1	0.988	0.973-1.004	0.3909
CpG2	0.993	0.982-1.004	0.6456
CpG3	0.993	0.987-0.999	0.0549
Adjusted for smoking			
CpG1	0.989	0.974-1.005	0.5685
CpG2	0.989	0.977-1.000	0.1812
CpG3	0.992	0.986-0.998	0.0177

Mixed effect Cox models, adjusted for age and sex, significance after Bonferroni correction, CI=confidence interval

Table S6. Study characteristics of the MIYoung study individuals selected for *GPR15* sequencing.

Characteristics	Controls	MI cases
n (%)	32	32
Females, n (%)	32 (100)	32 (100)
Age, years	75 (71-76)	42 (38-49)
Age at MI	NA	38 (34-39)
Cardiovascular risk factors		
Diabetes mellitus, n (%)	3 (9)	5 (16)
Body mass index, kg/m²	25 (24-28)	29 (27-33)
Hypertension, n (%)	15 (47)	18 (56)
Current smoker, n (%)	2 (6)	9 (28)
Family history of MI, n (%)	16 (50)	16 (50)
Lipid status		
LDL-cholesterol, mg/dl	113 (87-139)	87 (63-105)
HDL-cholesterol, mg/dl	55 (49-66)	45 (34-49)
LDL/HDL-ratio	1.8 (1.4-2.5)	2.0 (1.6-2.6)

Continuous variables are described by median values (25th percentile to 75th percentile). Dichotomous variables are presented as total numbers (%). HDL = high-density lipoprotein, LDL=low-density lipoprotein, MI=myocardial infarction, NA=data not available

Table S7. Associations between *GPR15* DNA methylation and rs2230344 minor T allele in the MIYoung study.

DNA methylation site	Effect	Standard error	p value
CpG1	1.157	0.748	0.3690
CpG2	0.690	1.289	1.7790
CpG3	2.846	1.356	0.1083
Adjusted for smoking			
CpG1	0.972	0.749	0.5850
CpG2	1.292	1.293	0.9540
CpG3	2.821	1.367	0.1182

Linear mixed models with CpG methylation status as dependent and rs2230344 allele dosage as independent variable, adjusted for age and sex, effect=% difference per T allele, CI=confidence interval

Table S8. Risk prediction of incident myocardial infarction by rs2230344 minor T allele in the FIA2 cohort.

Subjects	Hazard ratio	95% CI	p value
All	0.947	0.757-1.185	0.6352
Never smokers only	0.837	0.544-1.288	0.4191
Ever smokers only	1.044	0.801-1.360	0.7522

Mixed effect Cox models, adjusted for age and sex, CI=confidence interval

Table S9. Significantly differentially expressed genes between *Gpr15*$^{gfp/gfp}$ and wildtype mice in the myocardial infarction zone.

Table S9 is provided at the end of this file

Table S10. Cohort description.

	GIS [32-36]	MIYoung	FIA2 [37, 38]
Rationale	Discovery	Replication	Risk prediction
Study design	Restrospective case-control study	Retrospective case-control study	Prospective cohort/retrospective case-control study
Country	Germany	Germany	Sweden
Ethnicity	Caucasian	Caucasian	Caucasian
Cases	o Prevalent early onset MI<br>o n = 234	o Prevalent early onset MI<br>o n = 191	o Incident MI<br>o n = 295
Case definition	o Confirmed MI (positive ECG, increase in biomarkers of cardiac necrosis (troponin, creatine-kinase MB), or clinical symptoms) and proven by coronary angiography<br>o Age at MI ≤50 years<br>o Stable disease conditions for at least three months before study participation	o Confirmed MI (positive ECG, increase in biomarkers of cardiac necrosis (troponin, creatine-kinase MB), or clinical symptoms) and proven by coronary angiography<br>o Age at MI ≤50 years<br>o Stable disease conditions for at least three months before study participation	o MI events were identified through screening of hospital discharge records, general practitioners' reports, and death certificates.<br>o Age at MI = 34-71 years<br>o No prevalent MI cases
Eligible controls	o No manifest cardiovascular disease<br>o n = 419	o No manifest cardiovascular disease<br>o Age > 65 years<br>o n = 185	o No prevalent and incident MI<br>o n = 560
Matching criteria	o Sex<br>o Age	-	o Sex<br>o Age<br>o Date of health examination<br>o Geographical region
Number of controls per case	1	-	2
Number of matched samples for analyses	112 (112 cases, 112 controls)	-	170 (170 cases, 340 controls)
Samples	o Smoking (self-reported)<br>o RNA from PBMCs<br>o DNA from leukocytes	o Smoking (self-reported)<br>o RNA from PBMCs<br>o DNA from leukocytes	o Smoking (self-reported)<br>o DNA from whole blood

Ethical approval	local ethics committee of the Medical Chamber of Rhineland-Palatinate, Germany (ethical approval code 837.020.07 (5555))	local ethics committee of the University Medical Center Hamburg-Eppendorf, Germany (ethical approval code PV4137)	local ethics committee (Dnr 05-142M, with additional review in 2009-01-19)
Funding	Government of Rhineland-Palatinate ("Stiftung Rheinland-Pfalz für Innovation"), grant AZ 961-386261/733; "Wissen schafft Zukunft"; Johannes Gutenberg-University of Mainz "Center for Translational Vascular Biology"; Boehringer Ingelheimer, PHILIPS Medical Systems	-	Swedish Heart and Lung Foundation; Gustaf Sjölund Foundation; Country councils of Northern Sweden (Visare Norr) and the Faculty of Medicine of Umeå University

MI = myocardial infarction, PBMCs = peripheral blood mononuclear cells

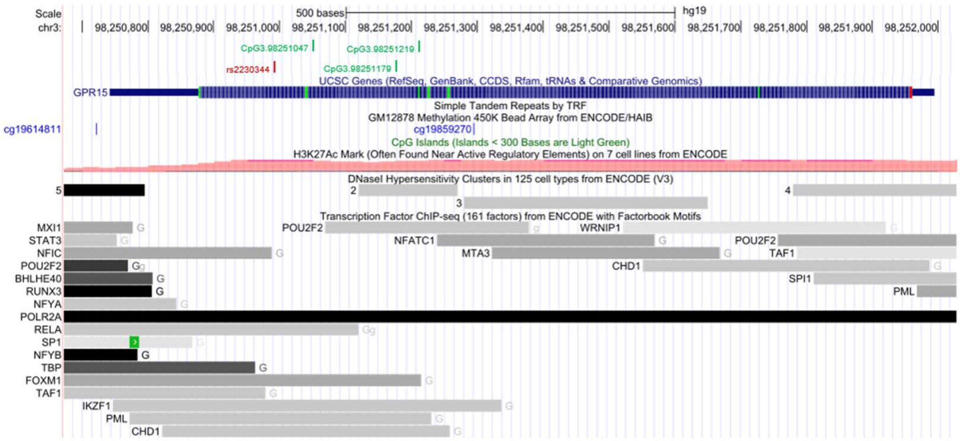

Figure S1. **The *GPR15* locus in the UCSC genome browser.** The *GPR15* locus depicted in UCSC Genome Browser on Human Feb. 2009 (GRCh37/hg19) Assembly, showing the *GPR15* gene, *GPR15* DNA methylation sites and rs2230344 SNP in close proximity to each other. http://genome.ucsc.edu/, 02/04/2020.

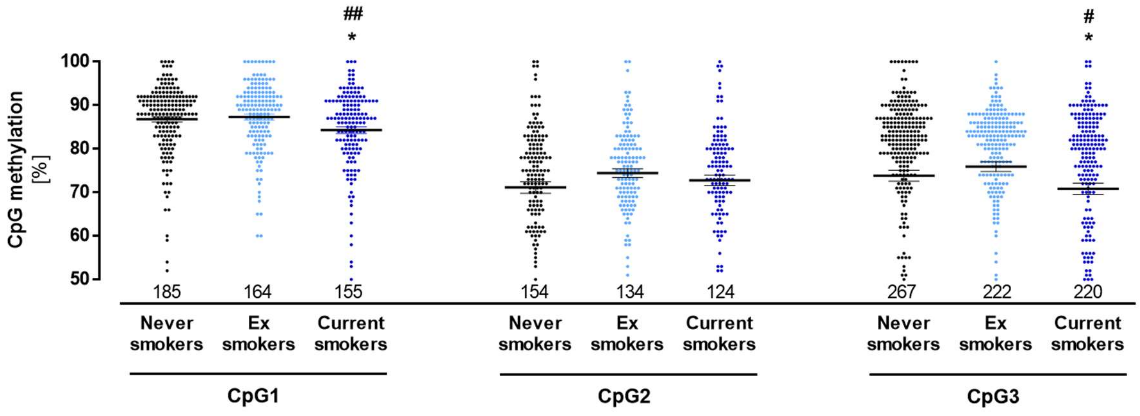

Figure S2. **Increased *GPR15* expression and decreased *GPR15* DNA methylation in smokers.** In the FIA2 study, *GPR15* DNA methylation was significantly lower in current smokers compared to never smokers and ex smokers for the CpG1 site ($p<0.05$ and $p<0.01$) and for the CpG3site ($p<0.05$). Kruskal-Wallis test with Dunns multiple comparison; significance threshold=0.0167 for Bonferroni correction, */#=$p<0.05$, ##=$p<0.01$ compared to the respective never smokers group/ ex smokers group; Data are shown as mean ± standard error of the mean.

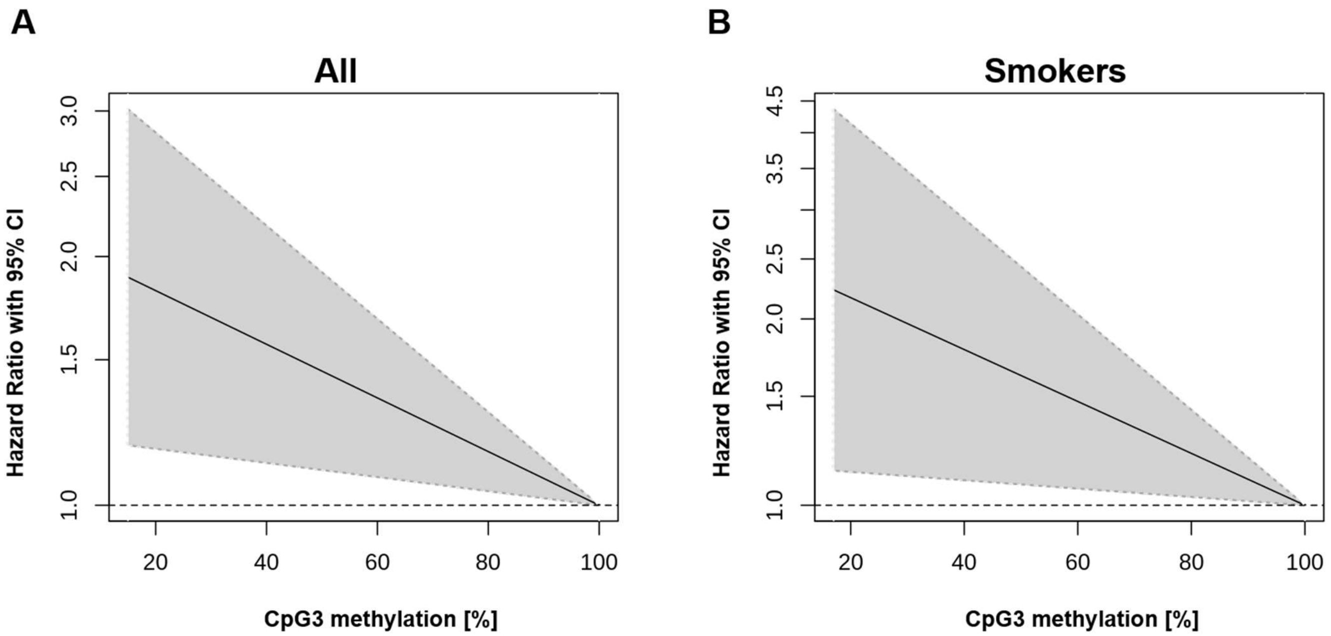

Figure S3. **Prediction of risk of incident myocardial infarction by *GPR15* DNA methylation in the FIA2 study.** CpG3 methylation significantly predicted the risk for incident MI after adjustment for smoking (HR=0.992, p=0.0177). MI risk was lower in individuals with higher CpG3 DNA methylation levels compared to individuals with lower CpG3 DNA methylation levels. This was shown in the overall cohort (**A**) as well as in smokers only (**B**). *R package smoothHR,* Full CpG3 site methylation (100%) was used as reference. Grey areas represent 95% confidence intervals (CI).

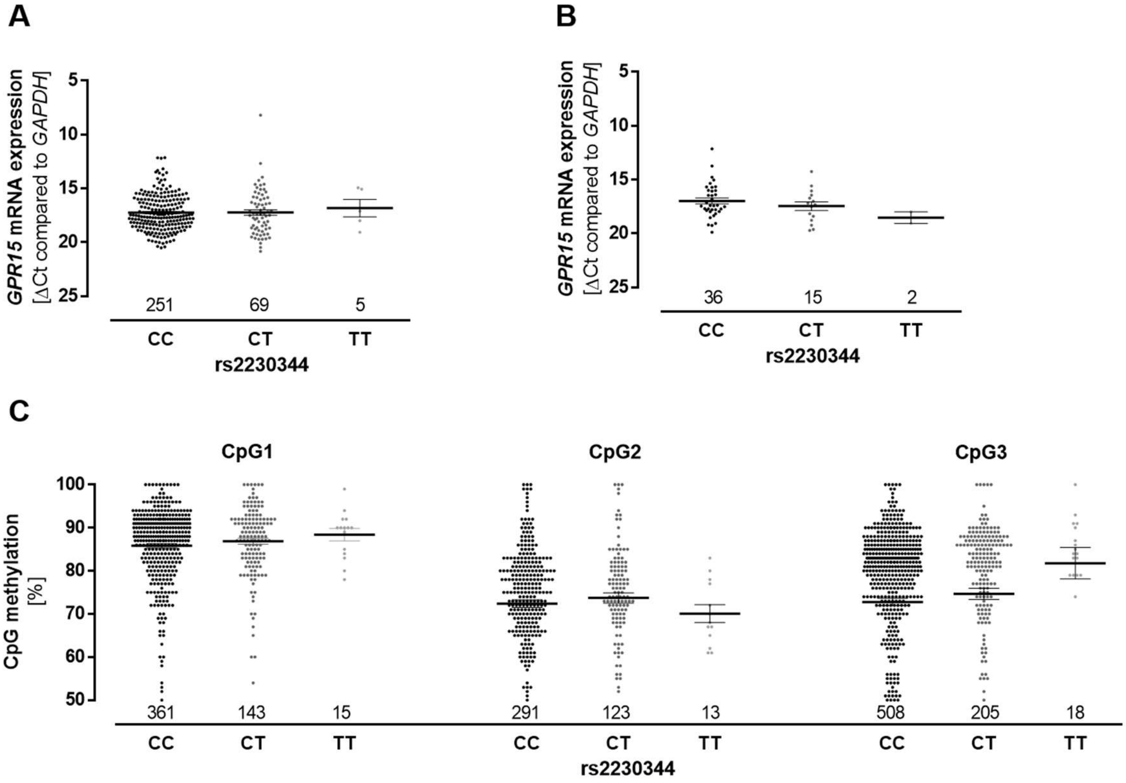

Figure S4. **rs2230344 genotype is not associated with *GPR15* DNA methylation or GPR15 mRNA expression. (A/B)** In the MIYoung study, rs2230344 SNP genotype was not significantly associated with *GPR15* mRNA expression in all individuals **(A)** or individuals with sequenced genotypes only **(B)**. *GPR15* mRNA expression is depicted as ΔCt values normalized to glyceraldehyde 3-phosphate dehydrogenase (*GAPDH*) mRNA expression. Lower ΔCt values indicate higher *GPR15* mRNA expression. **(C)** In the FIA2 study, *GPR15* DNA methylation of the CpG3 site was not significantly associated with rs2230344 genotype. n=13-508, linear mixed model, adjusted for age and sex; significance after Bonferroni correction Data are shown as mean±standard error of the mean.

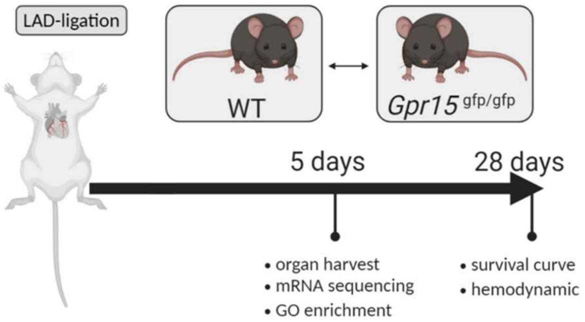

Figure S5. **Overview *Gpr15$^{gfp/gfp}$* mice.** *Gpr15$^{gfp/gfp}$* and wildtype (WT) mice were subjected to left anterior descending artery (LAD) ligation and either mRNA sequencing of the infarct zone (IZ) was performed 5 days after ligation or survival was determined 28 days after ligation.

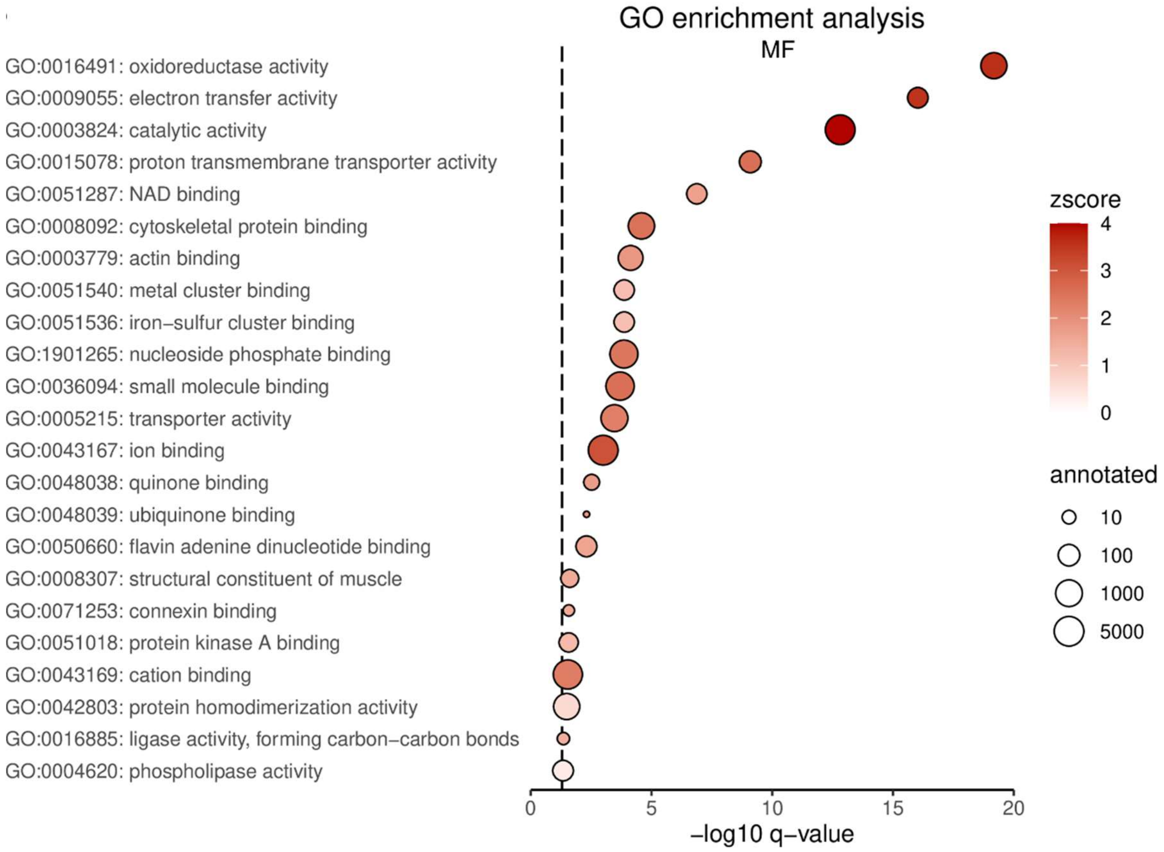

Figure S6. Altered GO terms for molecular functions in *Gpr15gfp/gfp* mice. Gene expression measured by 3′ mRNA sequencing of cardiac tissue from the IZ from 9 Bl6 wildtype (WT) and 8 *Gpr15gfp/gfp* mice 5 days after MI surgery. Gene Ontology (GO) terms are displayed for molecular functions (MF).

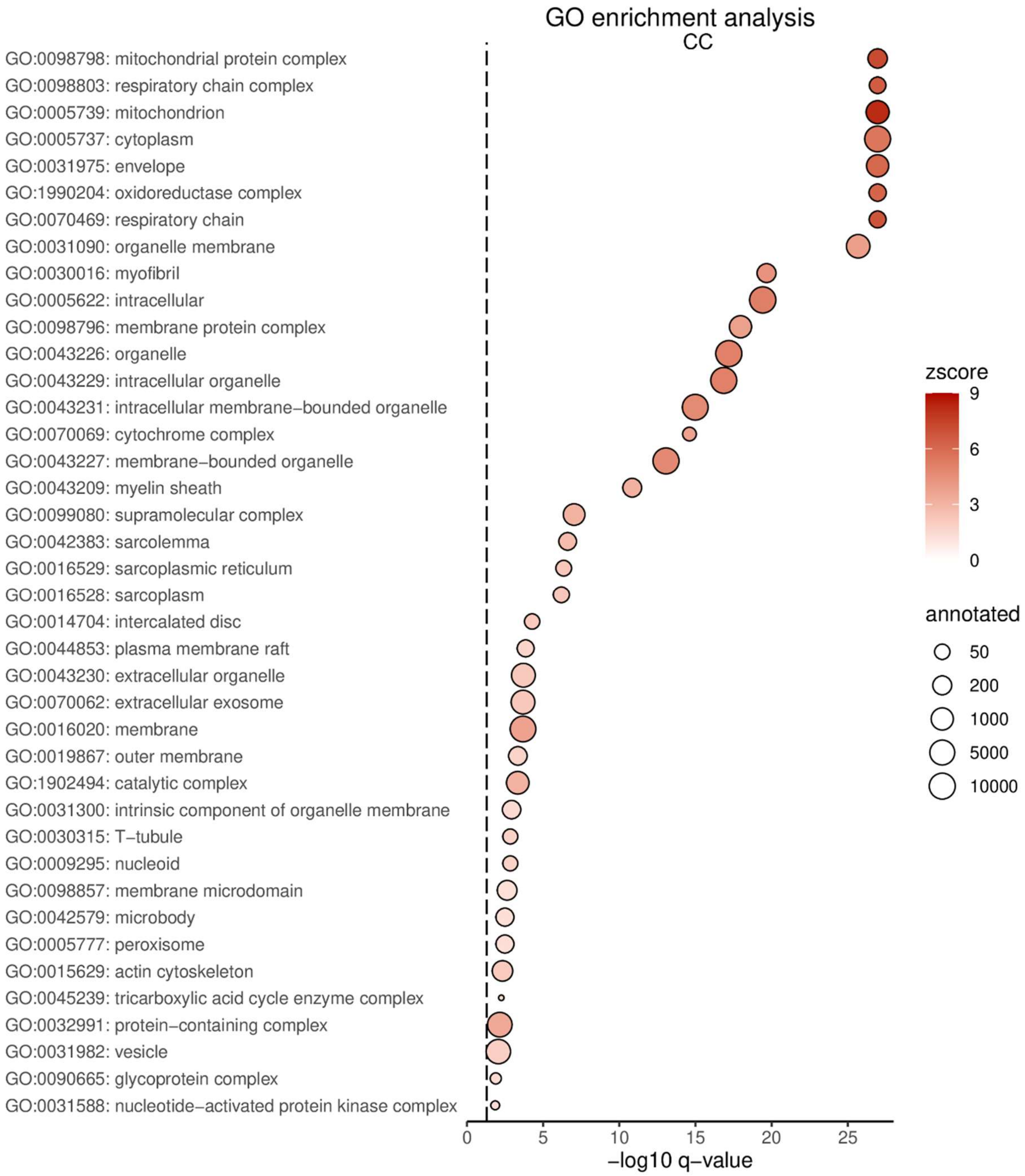

Figure S7. **Altered GO terms for cellular components in *Gpr15*$^{gfp/gfp}$ mice.** Gene expression measured by 3′ mRNA sequencing of cardiac tissue from the IZ from 9 Bl6 wildtype (WT) and 8 *Gpr15*$^{gfp/gfp}$ mice 5 days after MI surgery. Gene Ontology (GO) terms are displayed for cellular components (CC).

Table S2: Significantly differentially expressed genes between 112 early-onset MI patients and sex- and age-matched controls from the GIS discovery cohort.					
**gene**	**Ensembl_id**	**FC**	**p-value**	**q-value**	**gene description**
GPR15	ENSG00000154165	1,43E+00	1,87E-07	2,88E-03	G protein-coupled receptor 15
ABCA1	ENSG00000165029	-1,14E+00	4,54E-07	3,51E-03	ATP-binding cassette, sub-family A (ABC1), member 1
SELO	ENSG00000073169	-1,18E+00	1,35E-06	6,05E-03	selenoprotein O
CTF1	ENSG00000150281	1,15E+00	1,61E-06	6,05E-03	cardiotrophin 1
DUSP1	ENSG00000120129	-1,34E+00	2,19E-06	6,05E-03	dual specificity phosphatase 12 (EC:3.1.3.16 3.1.3.48)
DNM2	ENSG00000079805	-1,08E+00	2,35E-06	6,05E-03	dynamin 2 (EC:3.6.5.5)
DYNLL2	ENSG00000121083	-1,14E+00	4,05E-06	7,37E-03	dynein, light chain, LC8-type 2
CMIP	ENSG00000153815	-1,11E+00	4,25E-06	7,37E-03	CMIP
TMEM127	ENSG00000135956	-1,14E+00	4,29E-06	7,37E-03	transmembrane protein 127
GALT	ENSG00000213930	1,09E+00	5,36E-06	8,28E-03	galactose-1-phosphate uridylyltransferase (EC:2.7.7.12)
CLK3	ENSG00000179335	1,09E+00	7,80E-06	9,42E-03	CDC-like kinase 3 (EC:2.7.12.1)
IGF2R	ENSG00000197081	-1,09E+00	7,96E-06	9,42E-03	insulin-like growth factor 2 receptor
ADCY7	ENSG00000121281	-1,08E+00	8,00E-06	9,42E-03	adenylate cyclase 7 (EC:4.6.1.1)
ST6GALNAC3	ENSG00000184005	-1,13E+00	9,63E-06	9,42E-03	ST6
PIK3CD	ENSG00000171608	-1,06E+00	9,75E-06	9,42E-03	phosphoinositide-3-kinase, catalytic, delta polypeptide
MERTK	ENSG00000153208	-1,18E+00	1,03E-05	9,42E-03	c-mer proto-oncogene tyrosine kinase (EC:2.7.10.1)
SIPA1L1	ENSG00000197555	-1,12E+00	1,09E-05	9,42E-03	signal-induced proliferation-associated 1 like 1
HIP1R	ENSG00000130787	1,09E+00	1,10E-05	9,42E-03	huntingtin interacting protein 1 related
HDAC9	ENSG00000048052	1,08E+00	1,22E-05	9,90E-03	histone deacetylase 9 (EC:3.5.1.98)
C1orf38	ENSG00000130775	-1,17E+00	1,30E-05	1,01E-02	chromosome 1 open reading frame 38
SPRNP1	ENSG00000230786	-1,10E+00	1,46E-05	1,02E-02	NA
SPTAN1	ENSG00000197694	1,09E+00	1,51E-05	1,02E-02	spectrin, alpha, non-erythrocytic 1 (alpha-fodrin)
EPHA4	ENSG00000116106	1,13E+00	1,52E-05	1,02E-02	EPH receptor A4 (EC:2.7.10.1)
XXbac-B461K10.4	ENSG00000093100	1,09E+00	1,77E-05	1,07E-02	NA
OSGEP	ENSG00000092094	1,09E+00	1,80E-05	1,07E-02	O-sialoglycoprotein endopeptidase (EC:3.4.24.57)
BASP1	ENSG00000176788	-1,12E+00	1,80E-05	1,07E-02	brain abundant, membrane attached signal protein 1
SLC11A1	ENSG00000018280	-1,16E+00	2,57E-05	1,47E-02	solute carrier family 11 (proton-coupled divalent metal ion
PPP1R15B	ENSG00000158615	-1,09E+00	2,84E-05	1,57E-02	protein phosphatase 1, regulatory (inhibitor) subunit 15B
P2RY2	ENSG00000175591	-1,17E+00	2,94E-05	1,57E-02	purinergic receptor P2Y, G-protein coupled, 2
SNX12	ENSG00000147164	1,10E+00	3,08E-05	1,57E-02	sorting nexin 12
CD93	ENSG00000125810	-1,13E+00	3,16E-05	1,57E-02	CD93 molecule
MYO19	ENSG00000141140	1,07E+00	3,55E-05	1,71E-02	myosin XIX
VAMP7	ENSG00000124333	1,20E+00	4,03E-05	1,85E-02	vesicle-associated membrane protein 7
IL1R2	ENSG00000115590	-1,13E+00	4,19E-05	1,85E-02	interleukin 1 receptor, type II
IKZF3	ENSG00000161405	1,14E+00	4,20E-05	1,85E-02	IKAROS family zinc finger 3 (Aiolos)
CASK	ENSG00000147044	1,07E+00	4,50E-05	1,90E-02	calcium/calmodulin-dependent serine protein kinase (MAGUK family)
TNFAIP3	ENSG00000118503	-1,09E+00	4,54E-05	1,90E-02	tumor necrosis factor, alpha-induced protein 3
ADAM23	ENSG00000114948	1,13E+00	4,88E-05	1,98E-02	ADAM metallopeptidase domain 23
CYB5B	ENSG00000103018	1,10E+00	5,51E-05	2,13E-02	cytochrome b5 type B (outer mitochondrial membrane)
JARID2	ENSG00000008083	-1,07E+00	5,52E-05	2,13E-02	jumonji, AT rich interactive domain 2
SOAT1	ENSG00000057252	-1,11E+00	5,89E-05	2,22E-02	sterol O-acyltransferase 1 (EC:2.3.1.26)
AP000679.2	ENSG00000176984	1,25E+00	6,19E-05	2,26E-02	NA
STX6	ENSG00000135823	-1,07E+00	6,28E-05	2,26E-02	syntaxin 6
AC129778.1	ENSG00000230059	-1,10E+00	6,46E-05	2,27E-02	NA
U47924.25	ENSG00000237240	1,25E+00	6,84E-05	2,35E-02	NA
HIST1H2BK	ENSG00000197903	-1,15E+00	8,04E-05	2,70E-02	histone cluster 1, H2bk
PRKD3	ENSG00000115825	-1,08E+00	8,99E-05	2,96E-02	protein kinase D3 (EC:2.7.11.13)
TREM1	ENSG00000124731	-1,28E+00	9,31E-05	3,00E-02	triggering receptor expressed on myeloid cells 1
SPCS3	ENSG00000129128	1,16E+00	9,61E-05	3,03E-02	signal peptidase complex subunit 3 homolog (S. cerevisiae)
ALDH2	ENSG00000111275	-1,15E+00	9,90E-05	3,03E-02	aldehyde dehydrogenase 2 family (mitochondrial) (EC:1.2.1.3)
TNFAIP2	ENSG00000185215	-1,13E+00	9,99E-05	3,03E-02	tumor necrosis factor, alpha-induced protein 2
ARAP1	ENSG00000186635	-1,08E+00	1,02E-04	3,04E-02	ArfGAP with RhoGAP domain, ankyrin repeat and PH domain 1
MAFK	ENSG00000198517	-1,10E+00	1,08E-04	3,14E-02	v-maf musculoaponeurotic fibrosarcoma oncogene homolog K (avian)
RPL23	ENSG00000125691	1,16E+00	1,10E-04	3,14E-02	mitochondrial ribosomal protein L23
EXOSC1	ENSG00000171311	1,09E+00	1,21E-04	3,36E-02	exosome component 1
NELL2	ENSG00000184613	1,15E+00	1,22E-04	3,36E-02	NEL-like 2 (chicken)
FAM122A	ENSG00000187866	1,11E+00	1,29E-04	3,51E-02	family with sequence similarity 122A
WDR49	ENSG00000174776	-1,09E+00	1,34E-04	3,57E-02	WD repeat domain 49
MYLIP	ENSG00000007944	-1,08E+00	1,42E-04	3,71E-02	myosin regulatory light chain interacting protein (EC:6.3.2.-)
BCKDHB	ENSG00000083123	-1,11E+00	1,49E-04	3,75E-02	branched chain keto acid dehydrogenase E1, beta polypeptide
NA	ENSG00000188666	-1,14E+00	1,56E-04	3,75E-02	NA
LRRC70	ENSG00000186105	-1,14E+00	1,56E-04	3,75E-02	leucine rich repeat containing 70
PGS1	ENSG00000087157	-1,07E+00	1,57E-04	3,75E-02	phosphatidylglycerophosphate synthase 1 (EC:2.7.8.5)
CXorf65	ENSG00000204165	1,13E+00	1,57E-04	3,75E-02	chromosome X open reading frame 65
C6orf52	ENSG00000137434	-1,11E+00	1,58E-04	3,75E-02	chromosome 6 open reading frame 52
DTWD2	ENSG00000169570	1,09E+00	1,68E-04	3,94E-02	DTW domain containing 2
CTSZ	ENSG00000101160	-1,09E+00	1,77E-04	4,04E-02	cathepsin Z (EC:3.4.18.1)
ZSWIM6	ENSG00000130449	-1,10E+00	1,78E-04	4,04E-02	zinc finger, SWIM-type containing 6
RNF145	ENSG00000145860	-1,08E+00	1,85E-04	4,15E-02	ring finger protein 145
MAK	ENSG00000111837	-1,10E+00	2,03E-04	4,42E-02	male germ cell-associated kinase (EC:2.7.11.22)
PIGM	ENSG00000143315	-1,09E+00	2,03E-04	4,42E-02	phosphatidylinositol glycan anchor biosynthesis, class M
AMFR	ENSG00000159461	-1,11E+00	2,07E-04	4,45E-02	autocrine motility factor receptor (EC:6.3.2.19)
MIOS	ENSG00000164654	-1,08E+00	2,26E-04	4,67E-02	missing oocyte, meiosis regulator, homolog (Drosophila)
CRISPLD2	ENSG00000103196	-1,18E+00	2,26E-04	4,67E-02	cysteine-rich secretory protein LCCL domain containing 2
SERPINE2	ENSG00000135919	-1,09E+00	2,27E-04	4,67E-02	serpin peptidase inhibitor, clade E (nexin, plasminogen activator
BMP6	ENSG00000153162	-1,15E+00	2,36E-04	4,80E-02	bone morphogenetic protein 6
KIAA0930	ENSG00000100364	-1,09E+00	2,45E-04	4,88E-02	NA
HINT1	ENSG00000169567	1,13E+00	2,47E-04	4,88E-02	histidine triad nucleotide binding protein 1 (EC:3.-.-.-)
C7orf66	ENSG00000205174	1,12E+00	2,50E-04	4,88E-02	chromosome 7 open reading frame 66
DUS4L	ENSG00000105865	-1,09E+00	2,64E-04	5,07E-02	dihydrouridine synthase 4-like (S. cerevisiae)
KLHL24	ENSG00000114796	-1,08E+00	2,66E-04	5,07E-02	kelch-like 24 (Drosophila)
GALNT2	ENSG00000143641	-1,06E+00	2,70E-04	5,09E-02	UDP-N-acetyl-alpha-D-galactosamine:polypeptide
RNF125	ENSG00000101695	1,10E+00	2,79E-04	5,20E-02	ring finger protein 125
STX11	ENSG00000135604	-1,14E+00	2,87E-04	5,25E-02	syntaxin 11
AGER	ENSG00000204305	-1,08E+00	2,89E-04	5,25E-02	advanced glycosylation end product-specific receptor
HSPB1	ENSG00000106211	1,10E+00	2,92E-04	5,25E-02	heat shock 27kDa protein 1
ARF4	ENSG00000168374	1,15E+00	3,03E-04	5,34E-02	ADP-ribosylation factor 4
TMEM116	ENSG00000198270	1,11E+00	3,04E-04	5,34E-02	transmembrane protein 116
TNFRSF21	ENSG00000146072	-1,09E+00	3,18E-04	5,52E-02	tumor necrosis factor receptor superfamily, member 21
MYH13	ENSG00000006788	1,07E+00	3,23E-04	5,54E-02	myosin, heavy chain 13, skeletal muscle
TSC22D3	ENSG00000157514	-1,06E+00	3,28E-04	5,57E-02	TSC22 domain family, member 3
WDFY1	ENSG00000085449	-1,08E+00	3,40E-04	5,66E-02	WD repeat and FYVE domain containing 1
RALGPS1	ENSG00000136828	1,06E+00	3,40E-04	5,66E-02	Ral GEF with PH domain and SH3 binding motif 1
ITGB7	ENSG00000139626	1,09E+00	3,49E-04	5,71E-02	integrin, beta 7
NDUFV2	ENSG00000178127	1,16E+00	3,51E-04	5,71E-02	NADH dehydrogenase (ubiquinone) flavoprotein 2, 24kDa (EC:1.6.5.3

OPRD1	ENSG00000116329	-1,11E+00	3,58E-04	5,77E-02	opioid receptor, delta 1
SPINK8	ENSG00000229453	-1,13E+00	3,63E-04	5,79E-02	serine peptidase inhibitor, Kazal type 8 (putative)
MYCL1	ENSG00000116990	-1,10E+00	3,70E-04	5,84E-02	v-myc myelocytomatosis viral oncogene homolog 1, lung carcinoma
ABCD2	ENSG00000173208	1,10E+00	4,16E-04	6,32E-02	ATP-binding cassette, sub-family D (ALD), member 2
STX2	ENSG00000111450	1,13E+00	4,18E-04	6,32E-02	syntaxin 2
MAP3K6	ENSG00000142733	1,06E+00	4,21E-04	6,32E-02	mitogen-activated protein kinase kinase kinase 6 (EC:2.7.11.25)
PLCB4	ENSG00000101333	-1,07E+00	4,21E-04	6,32E-02	phospholipase C, beta 4 (EC:3.1.4.11)
DOK3	ENSG00000146094	-1,13E+00	4,28E-04	6,32E-02	docking protein 3
MGEA5	ENSG00000198408	1,11E+00	4,33E-04	6,32E-02	meningioma expressed antigen 5 (hyaluronidase) (EC:3.2.1.52
MTO1	ENSG00000135297	1,08E+00	4,34E-04	6,32E-02	mitochondrial translation optimization 1 homolog (S. cerevisiae)
SH2B3	ENSG00000111252	-1,06E+00	4,37E-04	6,32E-02	SH2B adaptor protein 3
UNC93B1	ENSG00000110057	-1,11E+00	4,47E-04	6,32E-02	unc-93 homolog B1 (C. elegans)
C19orf28	ENSG00000161091	-1,07E+00	4,53E-04	6,32E-02	chromosome 19 open reading frame 28
VSTM1	ENSG00000189068	-1,18E+00	4,57E-04	6,32E-02	V-set and transmembrane domain containing 1
SLC6A6	ENSG00000131389	-1,12E+00	4,59E-04	6,32E-02	solute carrier family 6 (neurotransmitter transporter, taurine),
ESRP1	ENSG00000104413	-1,08E+00	4,62E-04	6,32E-02	epithelial splicing regulatory protein 1
PTRF	ENSG00000177469	1,10E+00	4,67E-04	6,32E-02	polymerase I and transcript release factor
IL23A	ENSG00000110944	1,07E+00	4,68E-04	6,32E-02	interleukin 23, alpha subunit p19
STS	ENSG00000101846	-1,10E+00	4,72E-04	6,32E-02	steroid sulfatase (microsomal), isozyme S (EC:3.1.6.2)
PCYT1B	ENSG00000102230	-1,11E+00	4,74E-04	6,32E-02	phosphate cytidylyltransferase 1, choline, beta (EC:2.7.7.15)
MCL1	ENSG00000143384	-1,12E+00	4,74E-04	6,32E-02	myeloid cell leukemia sequence 1 (BCL2-related)
CDK5RAP1	ENSG00000101391	1,07E+00	4,92E-04	6,48E-02	CDK5 regulatory subunit associated protein 1
TRPS1	ENSG00000104447	-1,10E+00	4,98E-04	6,48E-02	trichorhinophalangeal syndrome I
ZNF812	ENSG00000224689	1,15E+00	4,99E-04	6,48E-02	zinc finger protein 812
DAPK1	ENSG00000196730	-1,11E+00	5,03E-04	6,48E-02	death-associated protein kinase 1 (EC:2.7.11.1)
TMEM110	ENSG00000213533	-1,10E+00	5,14E-04	6,51E-02	transmembrane protein 110
TMEM110-MUSTN1	ENSG00000248592	-1,10E+00	5,14E-04	6,51E-02	NA
JAZF1	ENSG00000153814	1,09E+00	5,24E-04	6,58E-02	JAZF zinc finger 1
HSPA2	ENSG00000126803	1,10E+00	5,43E-04	6,77E-02	heat shock 70kDa protein 2
POGK	ENSG00000143157	-1,07E+00	5,62E-04	6,95E-02	pogo transposable element with KRAB domain
HIST2H2BE	ENSG00000184678	-1,26E+00	5,71E-04	7,00E-02	histone cluster 2, H2be
NA	ENSG00000141325	1,07E+00	5,83E-04	7,05E-02	NA
SLC43A2	ENSG00000167703	-1,11E+00	5,84E-04	7,05E-02	solute carrier family 43, member 2
MCF2L	ENSG00000126217	1,05E+00	5,98E-04	7,15E-02	MCF.2 cell line derived transforming sequence-like
ATF7IP2	ENSG00000166669	1,13E+00	6,02E-04	7,15E-02	activating transcription factor 7 interacting protein 2
ABCG1	ENSG00000160179	-1,08E+00	6,06E-04	7,15E-02	ATP-binding cassette, sub-family G (WHITE), member 1
OR52J3	ENSG00000205495	1,16E+00	6,22E-04	7,28E-02	olfactory receptor, family 52, subfamily J, member 3
ASB13	ENSG00000196372	1,13E+00	6,34E-04	7,37E-02	ankyrin repeat and SOCS box-containing 13
AGTRAP	ENSG00000177674	-1,10E+00	6,42E-04	7,41E-02	angiotensin II receptor-associated protein
ETAA1	ENSG00000143971	1,11E+00	6,58E-04	7,54E-02	Ewing tumor-associated antigen 1
CXCL16	ENSG00000161921	-1,18E+00	6,67E-04	7,58E-02	chemokine (C-X-C motif) ligand 16
SLC40A1	ENSG00000138449	-1,14E+00	6,80E-04	7,63E-02	solute carrier family 40 (iron-regulated transporter), member 1
PPM1G	ENSG00000115241	1,07E+00	6,81E-04	7,63E-02	protein phosphatase, Mg2+/Mn2+ dependent, 1G (EC:3.1.3.16)
C19orf18	ENSG00000177025	1,14E+00	6,97E-04	7,74E-02	chromosome 19 open reading frame 18
PTGER2	ENSG00000125384	1,12E+00	7,05E-04	7,78E-02	prostaglandin E receptor 2 (subtype EP2), 53kDa
CCDC77	ENSG00000120647	-1,11E+00	7,18E-04	7,87E-02	coiled-coil domain containing 77
PRKAG2	ENSG00000106617	-1,06E+00	7,27E-04	7,91E-02	protein kinase, AMP-activated, gamma 2 non-catalytic subunit
RELA	ENSG00000173039	1,09E+00	7,64E-04	8,26E-02	v-rel reticuloendotheliosis viral oncogene homolog A (avian)
SGMS2	ENSG00000164023	-1,10E+00	7,92E-04	8,50E-02	sphingomyelin synthase 2 (EC:2.7.8.27)
ZDHHC18	ENSG00000204160	1,06E+00	8,10E-04	8,63E-02	zinc finger, DHHC-type containing 18
AC008021.1	ENSG00000206043	-1,15E+00	8,21E-04	8,69E-02	NA
C2CD3	ENSG00000168014	1,05E+00	8,51E-04	8,95E-02	C2 calcium-dependent domain containing 3
EDEM3	ENSG00000116406	-1,08E+00	8,62E-04	8,97E-02	ER degradation enhancer, mannosidase alpha-like 3
RIPK3	ENSG00000129465	1,08E+00	8,80E-04	8,97E-02	receptor-interacting serine-threonine kinase 3 (EC:2.7.11.1)
ST14	ENSG00000149418	-1,08E+00	8,85E-04	8,97E-02	suppression of tumorigenicity 14 (colon carcinoma) (EC:3.4.21.109)
IRF2BP2	ENSG00000168264	-1,07E+00	8,92E-04	8,97E-02	interferon regulatory factor 2 binding protein 2
CD300A	ENSG00000167851	-1,13E+00	8,94E-04	8,97E-02	CD300a molecule
USP14	ENSG00000101557	1,07E+00	9,10E-04	8,97E-02	ubiquitin specific peptidase 14 (tRNA-guanine transglycosylase)
CCDC56	ENSG00000183978	1,23E+00	9,10E-04	8,97E-02	coiled-coil domain containing 56
MARK1	ENSG00000116141	1,08E+00	9,12E-04	8,97E-02	MAP/microtubule affinity-regulating kinase 1 (EC:2.7.11.1)
C5orf17	ENSG00000248874	1,11E+00	9,19E-04	8,97E-02	NA
MICAL3	ENSG00000243156	1,08E+00	9,20E-04	8,97E-02	microtubule associated monoxygenase, calponin and LIM domain
EIF4ENIF1	ENSG00000184708	1,05E+00	9,25E-04	8,97E-02	eukaryotic translation initiation factor 4E nuclear import factor 1
ECE1	ENSG00000117298	-1,08E+00	9,27E-04	8,97E-02	endothelin converting enzyme 1 (EC:3.4.24.71)
INPP5D	ENSG00000168918	-1,06E+00	9,29E-04	8,97E-02	inositol polyphosphate-5-phosphatase, 145kDa
POU3F4	ENSG00000196767	1,10E+00	9,37E-04	8,99E-02	POU class 3 homeobox 4
MRO	ENSG00000134042	-1,09E+00	9,46E-04	8,99E-02	maestro
RP11-698N11.3	ENSG00000186082	1,11E+00	9,48E-04	8,99E-02	NA
NKX3-1	ENSG00000167034	1,07E+00	9,58E-04	9,03E-02	NK3 homeobox 1
DDX19A	ENSG00000168872	1,08E+00	9,78E-04	9,11E-02	DEAD (Asp-Glu-Ala-As) box polypeptide 19A
LDLR	ENSG00000130164	1,09E+00	9,78E-04	9,11E-02	low density lipoprotein receptor
CPA4	ENSG00000128510	-1,08E+00	9,85E-04	9,12E-02	carboxypeptidase A4
DEPDC5	ENSG00000100150	1,05E+00	1,00E-03	9,21E-02	DEP domain containing 5
SLC26A8	ENSG00000112053	-1,08E+00	1,02E-03	9,26E-02	solute carrier family 26, member 8
ROMO1	ENSG00000125995	1,15E+00	1,02E-03	9,26E-02	reactive oxygen species modulator 1
PARP6	ENSG00000137817	1,05E+00	1,04E-03	9,40E-02	poly (ADP-ribose) polymerase family, member 6 (EC:2.4.2.30)
ARHGAP17	ENSG00000140750	1,07E+00	1,06E-03	9,42E-02	Rho GTPase activating protein 17
MARVELD1	ENSG00000155254	-1,10E+00	1,06E-03	9,42E-02	MARVEL domain containing 1
RAP2B	ENSG00000181467	-1,09E+00	1,06E-03	9,42E-02	RAP2B, member of RAS oncogene family
AC027763.2	ENSG00000215067	-1,08E+00	1,07E-03	9,42E-02	NA
HBM	ENSG00000206177	1,15E+00	1,07E-03	9,42E-02	hemoglobin, mu
SERHL2	ENSG00000183569	1,11E+00	1,08E-03	9,42E-02	serine hydrolase-like 2
ALG12	ENSG00000182858	-1,06E+00	1,11E-03	9,66E-02	asparagine-linked glycosylation 12, alpha-1,6-mannosyltransferase
PLCG1	ENSG00000124181	1,08E+00	1,12E-03	9,66E-02	phospholipase C, gamma 1 (EC:3.1.4.11)
MAP3K9	ENSG00000006432	1,06E+00	1,13E-03	9,66E-02	mitogen-activated protein kinase kinase kinase 9 (EC:2.7.11.25)
CLIC5	ENSG00000112782	1,09E+00	1,14E-03	9,72E-02	chloride intracellular channel 5
RASGEF1A	ENSG00000198915	1,06E+00	1,15E-03	9,75E-02	RasGEF domain family, member 1A
CDK20	ENSG00000156345	1,08E+00	1,18E-03	9,95E-02	cyclin-dependent kinase 20 (EC:2.7.11.22)

Table S2: Differential gene expression in PBMCs between the sex and age- matched groups was identified by applying a linear mixed model.
The model was adjusted for the classical cardiovascular risk factors hypertension, smoking status, body mass index, diabetes and low-density lipoprotein/high-density lipoprotein ratio
n=112, FC=fold change, MI=myocardial infarction

**Table S9: Significantly differentially expressed genes between *Gpr15*$^{gfp/gfp}$ and wildtype mice in the myocardial infarction zone.**				
**gene**	**Ensembl_id**	**log2FC**	**p-value**	**gene description**
Muc16	ENSMUSG00000109564	3,47E+00	1,65E-04	mucin 16
Cxcl14	ENSMUSG00000021508	1,03E+00	6,49E-04	chemokine (C-X-C motif) ligand 14
Mertk	ENSMUSG00000014361	6,51E-01	8,18E-04	c-mer proto-oncogene tyrosine kinase
Abcb4	ENSMUSG00000042476	-1,48E+00	5,27E-06	ATP-binding cassette, sub-family B (MDR/TAP), member 4
Fam162a	ENSMUSG00000003955	-6,43E-01	5,43E-06	family with sequence similarity 162, member A
E230013L22Rik	ENSMUSG00000096957	-1,59E+00	4,31E-05	RIKEN cDNA E230013L22 gene
Slc16a1	ENSMUSG00000032902	-1,09E+00	4,43E-05	solute carrier family 16 (monocarboxylic acid transporters), member 1
4631405J19Rik	ENSMUSG00000075027	-1,74E+00	4,91E-05	RIKEN cDNA 4631405J19 gene
Kcnj12	ENSMUSG00000042529	-1,55E+00	7,08E-05	potassium inwardly-rectifying channel, subfamily J, member 12
Cap2	ENSMUSG00000021373	-8,27E-01	8,05E-05	CAP, adenylate cyclase-associated protein, 2 (yeast)
Ndufaf6	ENSMUSG00000050323	-8,96E-01	9,27E-05	NADH dehydrogenase (ubiquinone) complex I, assembly factor 6
Fgf13	ENSMUSG00000031137	-1,20E+00	9,58E-05	fibroblast growth factor 13
Krt222	ENSMUSG00000035849	-1,71E+00	9,72E-05	keratin 222
Ldhd	ENSMUSG00000031958	-1,11E+00	1,16E-04	lactate dehydrogenase D
Cacnb2	ENSMUSG00000057914	-8,32E-01	1,17E-04	calcium channel, voltage-dependent, beta 2 subunit
Slc25a33	ENSMUSG00000028982	-9,66E-01	1,26E-04	solute carrier family 25, member 33
Tmem74b	ENSMUSG00000044364	-2,62E+00	1,31E-04	transmembrane protein 74B
Gm6123	ENSMUSG00000103922	-1,92E+00	1,33E-04	predicted gene 6123
Pip5k1b	ENSMUSG00000024867	-1,07E+00	1,37E-04	phosphatidylinositol-4-phosphate 5-kinase, type 1 beta
Akap1	ENSMUSG00000018428	-8,07E-01	1,43E-04	A kinase (PRKA) anchor protein 1
Gm17546	ENSMUSG00000078648	-3,21E+00	1,55E-04	predicted gene, 17546
Lgr6	ENSMUSG00000042793	-1,31E+00	1,61E-04	leucine-rich repeat-containing G protein-coupled receptor 6
Gm11716	ENSMUSG00000086298	-1,12E+00	1,64E-04	predicted gene 11716
Acss2	ENSMUSG00000027605	-8,16E-01	1,66E-04	acyl-CoA synthetase short-chain family member 2
Cdkl2	ENSMUSG00000029403	-9,42E-01	1,88E-04	cyclin-dependent kinase-like 2 (CDC2-related kinase)
0610040B10Rik	ENSMUSG00000089889	-1,05E+00	1,98E-04	RIKEN cDNA 0610040B10 gene
Ube2ql1	ENSMUSG00000052981	-1,21E+00	2,04E-04	ubiquitin-conjugating enzyme E2Q family-like 1
Grb14	ENSMUSG00000026888	-9,15E-01	2,04E-04	growth factor receptor bound protein 14
Kbtbd12	ENSMUSG00000033182	-1,23E+00	2,14E-04	kelch repeat and BTB (POZ) domain containing 12
Fhl2	ENSMUSG00000008136	-1,16E+00	2,25E-04	four and a half LIM domains 2
Pygo1	ENSMUSG00000034910	-8,24E-01	2,39E-04	pygopus 1
Atp5s	ENSMUSG00000054894	-7,28E-01	2,69E-04	ATP synthase, H+ transporting, mitochondrial F0 complex, subunit S
Bves	ENSMUSG00000071317	-1,07E+00	2,84E-04	blood vessel epicardial substance
Cox7c	ENSMUSG00000017778	-6,95E-01	3,05E-04	cytochrome c oxidase subunit VIIc
Smpx	ENSMUSG00000041476	-8,88E-01	3,08E-04	small muscle protein, X-linked
Tfrc	ENSMUSG00000022797	-1,28E+00	3,17E-04	transferrin receptor
Ppp1r3d	ENSMUSG00000049999	-8,52E-01	3,19E-04	protein phosphatase 1, regulatory subunit 3D
Rab3ip	ENSMUSG00000064181	-6,68E-01	3,19E-04	RAB3A interacting protein
Klhdc1	ENSMUSG00000051890	-8,62E-01	3,22E-04	kelch domain containing 1
Gm16399	ENSMUSG00000097790	-7,88E-01	3,37E-04	predicted pseudogene 16399
Tmem246	ENSMUSG00000039611	-9,89E-01	3,67E-04	transmembrane protein 246
Esrrb	ENSMUSG00000021255	-1,25E+00	4,13E-04	estrogen related receptor, beta
Mfn1	ENSMUSG00000027668	-8,03E-01	4,13E-04	mitofusin 1
Cox6c	ENSMUSG00000014313	-6,16E-01	4,18E-04	cytochrome c oxidase subunit VIc
Halr1	ENSMUSG00000085412	-3,17E+00	4,61E-04	Hoxa adjacent long noncoding RNA 1
Timp4	ENSMUSG00000030317	-2,10E+00	5,04E-04	tissue inhibitor of metalloproteinase 4
Armc2	ENSMUSG00000071324	-1,18E+00	5,10E-04	armadillo repeat containing 2
Grip2	ENSMUSG00000030098	-1,62E+00	5,23E-04	glutamate receptor interacting protein 2
Acat1	ENSMUSG00000032047	-7,39E-01	5,31E-04	acetyl-Coenzyme A acetyltransferase 1
Frmd5	ENSMUSG00000027238	-1,17E+00	5,56E-04	FERM domain containing 5
Gm43032	ENSMUSG00000106498	-3,01E+00	5,72E-04	predicted gene 43032
D3Ertd751e	ENSMUSG00000025766	-9,20E-01	5,79E-04	DNA segment, Chr 3, ERATO Doi 751, expressed
Egln1	ENSMUSG00000031987	-7,02E-01	5,79E-04	egl-9 family hypoxia-inducible factor 1
Usp28	ENSMUSG00000032267	-1,05E+00	6,16E-04	ubiquitin specific peptidase 28
Mipep-ps	ENSMUSG00000093624	-2,75E+00	6,21E-04	mitochondrial intermediate peptidase, pseudogene
D830039M14Rik	ENSMUSG00000043126	-1,79E+00	6,26E-04	RIKEN cDNA D830039M14 gene
Arhgef9	ENSMUSG00000025656	-9,91E-01	6,37E-04	CDC42 guanine nucleotide exchange factor (GEF) 9
Asb11	ENSMUSG00000031382	-1,48E+00	6,56E-04	ankyrin repeat and SOCS box-containing 11
CU074400.1	ENSMUSG00000114254	-9,36E-01	6,60E-04	NA
Gm16793	ENSMUSG00000097357	-2,37E+00	6,63E-04	predicted gene, 16793
Gm6238	ENSMUSG00000082996	-1,58E+00	6,71E-04	predicted pseudogene 6238
Cycs	ENSMUSG00000063694	-7,64E-01	6,76E-04	cytochrome c, somatic
Pdk1	ENSMUSG00000006494	-5,92E-01	6,94E-04	pyruvate dehydrogenase kinase, isoenzyme 1
2210408F21Rik	ENSMUSG00000087380	-7,06E-01	7,03E-04	RIKEN cDNA 2210408F21 gene
Ndufb2	ENSMUSG00000002416	-6,10E-01	7,26E-04	NADH dehydrogenase (ubiquinone) 1 beta subcomplex, 2
Gm10053	ENSMUSG00000058927	-7,19E-01	7,43E-04	predicted gene 10053
Eml6	ENSMUSG00000044072	-1,38E+00	7,44E-04	echinoderm microtubule associated protein like 6
Nlrp10	ENSMUSG00000049709	-1,08E+00	7,46E-04	NLR family, pyrin domain containing 10
Slc25a4	ENSMUSG00000031633	-9,71E-01	7,51E-04	solute carrier family 25 (mitochondrial carrier, adenine nucleotide translocator), member 4
Ppif	ENSMUSG00000021868	-1,06E+00	7,94E-04	peptidylprolyl isomerase F (cyclophilin F)
Gm26672	ENSMUSG00000097330	-2,20E+00	8,05E-04	predicted gene, 26672
Kcnd2	ENSMUSG00000060882	-1,79E+00	8,05E-04	potassium voltage-gated channel, Shal-related family, member 2
Gm17021	ENSMUSG00000091661	-1,40E+00	8,21E-04	predicted gene 17021
Pacsin3	ENSMUSG00000027257	-7,54E-01	8,45E-04	protein kinase C and casein kinase substrate in neurons 3
6430571L13Rik	ENSMUSG00000037977	-1,89E+00	8,60E-04	RIKEN cDNA 6430571L13 gene
Scn5a	ENSMUSG00000032511	-7,42E-01	8,71E-04	sodium channel, voltage-gated, type V, alpha
Dld	ENSMUSG00000020664	-6,52E-01	8,81E-04	dihydrolipoamide dehydrogenase
Lppos	ENSMUSG00000097867	-6,12E-01	8,86E-04	LIM domain containing preferred translocation partner in lipoma, opposite strand
Mterf2	ENSMUSG00000049038	-9,85E-01	9,28E-04	mitochondrial transcription termination factor 2
Cavin4	ENSMUSG00000028348	-1,32E+00	9,44E-04	caveolae associated 4
Mdh1	ENSMUSG00000020321	-8,58E-01	9,52E-04	malate dehydrogenase 1, NAD (soluble)
Trp53inp2	ENSMUSG00000038375	-6,80E-01	9,65E-04	transformation related protein 53 inducible nuclear protein 2
Acyp2	ENSMUSG00000060923	-7,28E-01	9,78E-04	acylphosphatase 2, muscle type
Nebl	ENSMUSG00000053702	-8,90E-01	9,82E-04	nebulette

Table S9: Differential cardiac gene expression between *Gpr15*$^{gfp/gfp}$ and wildtype mice was calculated using DESeq2. n=8/9, FC=fold change
